# Mechanochemical Synthesis and Magnetic Properties of the Mixed-Valent Binary Silver(I,II) Fluorides, Ag^I^_2_Ag^II^F_4_ and Ag^I^Ag^II^F_3_

**DOI:** 10.1021/jacs.4c11772

**Published:** 2024-10-24

**Authors:** Matic Belak Vivod, Zvonko Jagličić, Graham King, Thomas C. Hansen, Matic Lozinšek, Mirela Dragomir

**Affiliations:** † Jožef Stefan Institute, Jamova cesta 39, 1000 Ljubljana, Slovenia; ‡ Jožef Stefan International Postgraduate School, Jamova cesta 39, 1000 Ljubljana, Slovenia; § Institute of Mathematics, Physics and Mechanics, 1000 Ljubljana, Slovenia; ∥ Faculty of Civil and Geodetic Engineering, University of Ljubljana, Jamova cesta 2, 1000 Ljubljana, Slovenia; ⊥ Canadian Light Source, 44 Innovation Blvd, Saskatoon, S7N 2V3 Saskatchewan, Canada; # Institut Laue-Langevin, 38042 Grenoble Cedex 9, France

## Abstract

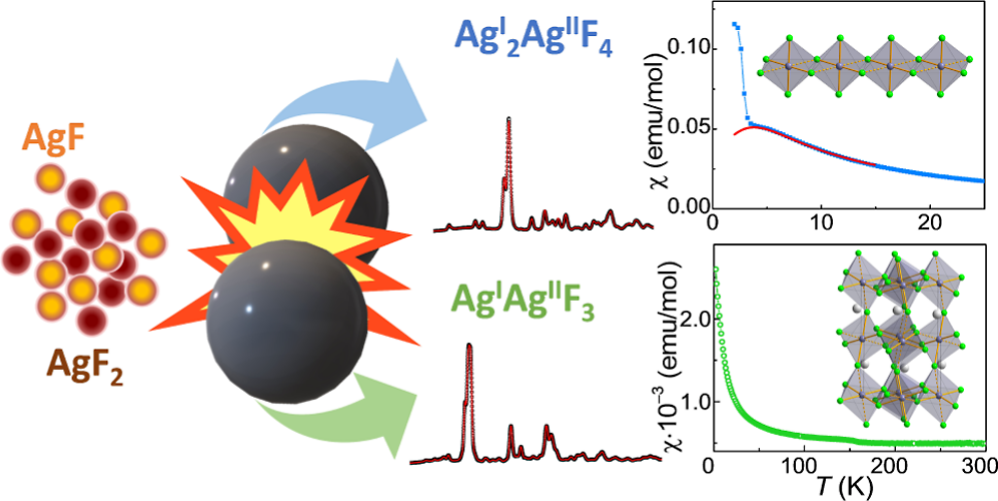

Fluoridoargentates(II) represent a fascinating class of silver(II) compounds with structural and magnetic similarities to cuprate superconductors. However, their synthesis is challenging, leaving their properties largely underexplored and hindering the discovery of new phases. This study introduces mechanochemistry as a novel approach for the synthesis of fluoridoargentates(II), avoiding the use of anhydrous HF or elemental fluorine and employing readily available equipment. Notably, ball milling of commercially available precursors successfully produced the long-sought-after first two examples of binary mixed-valent silver(I,II) phases, Ag^I^_2_Ag^II^F_4_ (Ag_3_F_4_) and Ag^I^Ag^II^F_3_ (Ag_2_F_3_). While the Ag^I^_2_Ag^II^F_4_ phase was obtained at room temperature, the Ag^I^Ag^II^F_3_ phase is metastable and required milling under cryogenic conditions. Characterization by synchrotron powder X-ray and neutron diffraction revealed that Ag^I^_2_Ag^II^F_4_ crystallizes in the *P*2_1_/*c* space group and is isostructural to β-K_2_AgF_4_. In this crystal structure, [Ag^II^F_2_F_4/2_]^2–^ distorted octahedral units with 4 + 2 coordination, extend parallel to *a*-crystallographic axis giving a quasi-one-dimensional canted antiferromagnetic character, as shown by magnetic susceptibility. The triclinic perovskite Ag^I^Ag^II^F_3_ phase adopts the *P*1̅ space group, is isostructural to AgCuF_3_ and also shows features of a one-dimensional antiferromagnet. This mechanochemical approach, also successfully applied to synthesize β-K_2_AgF_4_, is expected to expand the field of silver(II) chemistry, accelerating the search for silver analogs to cuprate superconductors and potentially extending to other cations in unusual oxidation states.

## Introduction

Systems containing *S* = 1/2 cations offer a rich playground to explore quantum effects such as spin liquids, spin-Peierls instabilities, superconductivity and the like.^1,2^ Compared to other metal cations like Cu^2+^, Ti^3+^, or V^4+^, systems containing Ag^2+^ are less explored although they show great promise for very intriguing physics such as exotic magnetism and even potential superconductivity as cuprate analogues.^3,4^ However, unlike Cu^2+^, which is in a common oxidation state, Ag^2+^ is an unstable and very reactive species.^5,6^ In oxides, Ag^2+^ disproportionates to Ag^+^ and Ag^3+^. Fluorine is the only element that is sufficiently electronegative to readily stabilize Ag in a +2 oxidation state. These compounds, fluoridoargentates(II), are truly exceptional in various aspects.^7^ Even the most fundamental binary compound, AgF_2_, displays intriguing structural and electronic properties similar to the parent compound of the cuprate superconductors, La_2_CuO_4_,^8^ which features strong mixing of Ag(4d^9^) and F(2p^2^) orbitals, a significant covalency of the Ag–F bond,^9^ and significant magnetic superexchange in two dimensions.^10^ Similar to Cu^2+^(3d^9^), the Ag^2+^(4d^9^), *S* = 1/2, can also be found in a Jahn–Teller elongated or compressed octahedral environment.

By analogy with cuprates, where superconductivity can be achieved by charge doping, mixed-valence silver(I,II) or silver(II,III) fluorides are very enticing as potential candidates for metallization analogous to charge-doped cuprates.^3^ However, synthesizing these phases has proven challenging, with silver(I,II) fluorides being particularly elusive. Currently, the binary fluorine–silver system only comprises six phases: Ag_2_F,^11−13^ AgF,^14,15^ AgF_2_,^16−18^ AgF_3_,^19,20^ Ag_2_F_5_,^21^ and Ag_3_F_8_.^22^ In these compounds, silver adopts the +1/2, +1, +2 and +3 oxidation states. Interestingly, only two binary mixed-valence silver fluorides are known up to date: Ag_2_F_5_ or Ag^II^F[Ag^III^F_4_], and Ag_3_F_8_ or Ag^II^[Ag^III^F_4_]_2_. In both cases, silver is in a mixed +2/+3 oxidation state. No binary Ag–F phases exist with silver in a mixed +1/+2 oxidation state. However, theoretical calculations have been performed to search for the most stable compounds and structures in the binary Ag–F system.^23−25^ Of outmost interest are mixed valence silver(I,II) binary fluorides, highly sought-after phases as they are believed to be the charged-doped AgF_2_ equivalents. These theoretical studies suggested that two novel stoichiometries with silver in a mixed +1/+2 oxidation state, namely Ag_3_F_4_ and Ag_2_F_3_, could be thermodynamically stable at ambient conditions. The hypothetical Ag^I^_2_Ag^II^F_4_ or Ag_3_F_4_ phase was predicted^23^ to adopt a monoclinic *P*2_1_/*c* Na_2_CuF_4_-type^26^ structure analogous to β-K_2_AgF_4_.^27^ A theoretical investigation of silver fluorides under pressure proposed a tetragonal *I*4̅2*d* CdMn_2_O_4_-type structure as the ground state structure of Ag^I^_2_Ag^II^F_4_ which was predicted to be stable up to 19 GPa.^24^ The existence of another silver(I,II) fluoride with the formula Ag^I^Ag^II^F_3_ or Ag_2_F_3_ was also predicted by density functional theory calculations. Three nearly energy-equivalent structure types were predicted: *P*1̅ (NaCuF_3_-type), *Pbnm* (KAgF_3_-type) and, *Pmcn* (CuTeO_3_-type structure).^23^ Another theoretical study proposed a structure with the *P*2_1_/*m* space group (distorted CaIrO_3_-type structure).^24^ The calculated energies of formation for the most stable polytype was −0.09 eV per formula unit for Ag^I^Ag^II^F_3_ and −0.21 eV for Ag^I^_2_Ag^II^F_4_.^23^ The more exothermic energy of formation for Ag^I^_2_Ag^II^F_4_ compared to Ag^I^Ag^II^F_3_ could indicate that Ag^I^_2_Ag^II^F_4_ is the most thermodynamically stable product of the reaction between AgF_2_ and AgF. Indeed, Ag_2_F_3_ was predicted to be only marginally stable with respect to decomposition into Ag_3_F_4_ and AgF_2_ at ambient pressure.^24^

Following these predictions, experimental efforts to synthesize these phases have fallen short, as current synthetic methods have not been able to provide access to these compounds which could be metastable. The main synthetic challenges were the poor solubility of the main precursor, AgF_2_, in anhydrous HF and thermal decomposition of AgF_2_ at temperatures <400 °C. Mechanochemistry could overcome the disadvantages of conventional synthesis methods, by offering room-temperature reaction conditions, enhanced kinetics, and solvent-free synthesis.^28−31^ Mechanochemistry represents a paradigm shift in the synthesis of materials, enabling the formation of metastable phases that are difficult to obtain using high-temperature methods. While there are numerous reports on the high-energy milling of oxides,^32^ indicating that it is an established synthetic technique, the mechanochemistry of fluorides on the other hand is still in its early stages of development,^33,34^ with the mechanochemistry of fluoridoargentates(II) being virtually an unexplored area of research at the onset of this work.

In this study, it is demonstrated that mechanochemistry is an effective approach to the formation of the anticipated mixed-valent binary silver(I,II) fluorides and showcases that it could be applied to the synthesis of fluoridoargentates(II) and other compounds with elements in unusual and mixed-valent oxidation states.

## Results and Discussion

### Ag^I^_2_Ag^II^F_4_

**Figure 1:**
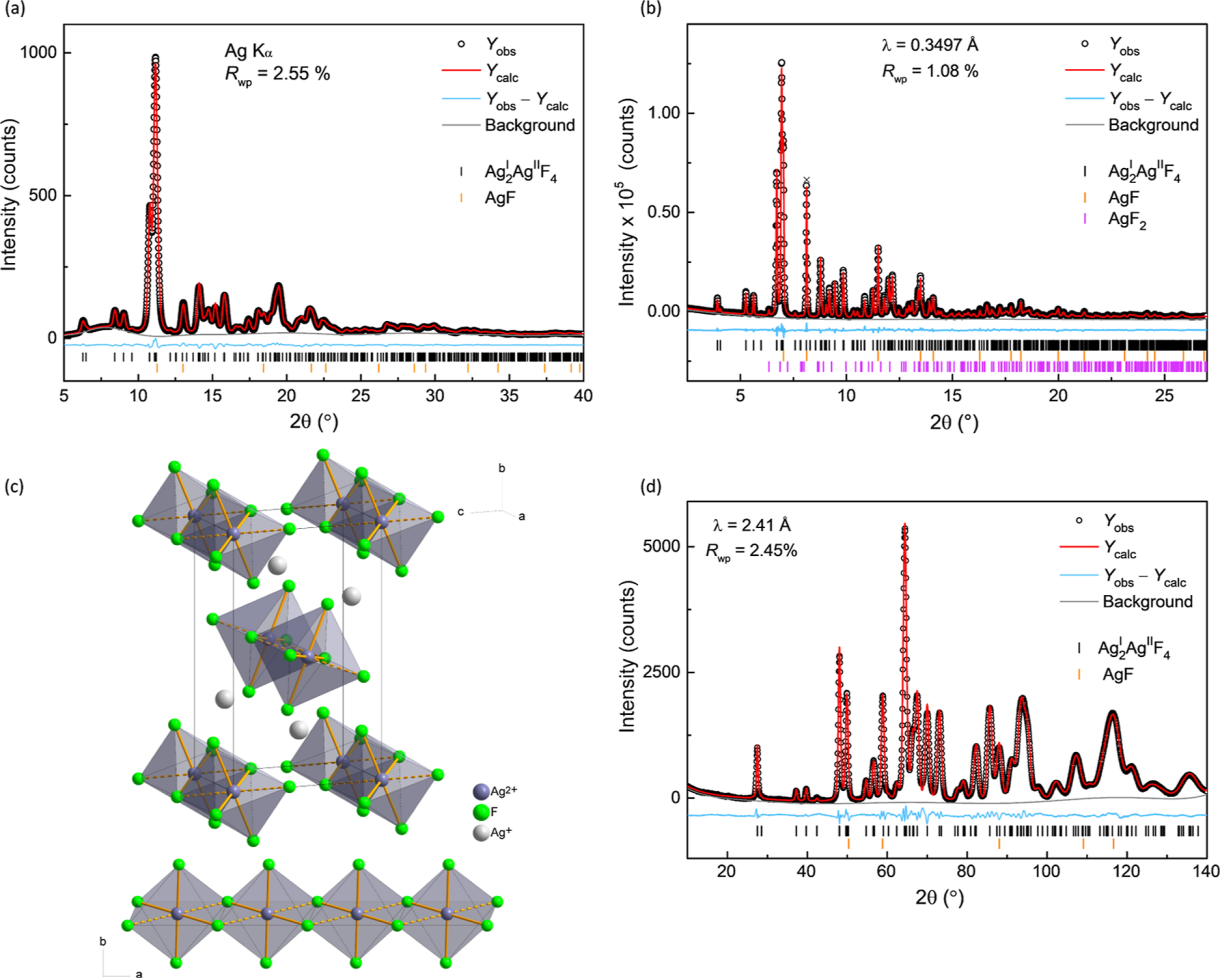
(a) Rietveld refinement of the laboratory PXRD data (Ag Kα radiation, λ = 0.5609 Å) of mechanochemically synthesized sample of Ag^I^_2_Ag^II^F_4_ collected at room temperature. Quantitative analysis: Ag^I^_2_Ag^II^F_4_: 88.7(2) wt %; AgF: 11.3(6) wt %. (b) Three-phase Rietveld refinement of the synchrotron data collected on the Ag^I^_2_Ag^II^F_4_ sample prepared by solid-state method. Quantitative analysis: Ag^I^_2_Ag^II^F_**4**_: 76.2(1) wt %; AgF: 22.9(1) wt %; AgF_2_: 0.9(5) wt %. (c) Unit cell of the Ag^I^_2_Ag^II^F_4_ (Ag_3_F_4_) crystal structure and a chain of edge-sharing [Ag^II^F_2_F_4/2_]^2–^ distorted octahedral units with 4 + 2 coordination, which extends parallel to *a*-crystallographic axis. (d) The 5 K NPD pattern of the ball-milled Ag^I^_2_Ag^II^F_4_ sample showing that the only impurity is small amounts of diamagnetic AgF. Quantitative analysis: Ag^I^_2_Ag^II^F_**4**_: 89.4(1) wt %; AgF: 10.6(1) wt %.

For the synthesis of Ag^I^_2_Ag^II^F_4_, a 2:1 AgF–AgF_2_ molar ratio was ball milled at room temperature. Experimental details are provided in the Supporting Information. A laboratory powder X-ray diffraction (PXRD) (λ = 0.5609 Å) analysis of the resulting pale light-brown polycrystalline sample confirmed the formation of the new Ag_3_F_4_ phase [88.7(2) wt %], as well as the presence of AgF [11.3(6) wt %] as a result of decomposition of AgF_2_ during milling (Figure 1a). After the successful mechanochemical synthesis, an attempt was made to synthesize Ag_3_F_4_ via a solid-state route, using a method similar to that employed for M_2_AgF_4_ (M = Na–Cs) compounds.^35^ However, to minimize the decomposition of AgF_2_, the optimized procedure involved a lower synthesis temperature (300 °C), which required longer dwell times of up to 3 days. A comparison of the laboratory diffractograms of the ball-milled and solid-state synthesized samples (Figure S1) reveals that, while both samples exhibit diffraction peaks belonging to the new compound, the solid-state sample contains more impurities. On the other side, the ball-milled sample shows significant peak broadening. This broadening suggests the presence of nanometer-sized crystallites and defects or microstrain induced during the milling process. A higher background is also observed in the milled sample, indicating the presence of an amorphous phase. Similar observations were also noted in the milling of oxides.^32^ Although the solid-state sample appeared to contain a larger amount of impurities than the ball-milled one, high-resolution synchrotron PXRD data (λ = 0.3497 Å) was collected at room temperature on the sample prepared by the solid-state method for indexation and structure refinement, due to its higher crystallinity (Figure 1b). Unassigned peaks that did not belong to unreacted reagents (AgF or AgF_2_) could be indexed to a monoclinic unit cell in the *P*2_1_/*c* space group (Figure S2) with unit cell parameters similar to Na_2_CuF_4_.^26^

A Rietveld refinement analysis performed using the Na_2_CuF_4_-structure type with a monoclinic *P*2_1_/*c* unit cell resulted in an excellent fit (Figure 1b) and the following unit cell parameters: *a* = 3.56910(8) Å, *b* = 9.8866(2) Å, *c* = 5.9829(2) Å, β = 92.832(2)°, *V* = 210.857(4) Å^3^, *Z* = 2 (Table S1a, Figures 1c and S3). The observed Ag^I^–F distances [2.391(7)–2.884(8) Å] and three sets of Ag^II^–F bond lengths [2.110(6), 2.119(7) and 2.529(7) Å] are in the expected range (Table S1b).^15,18,27,36^ The edge-sharing distorted octahedral [AgF_6_]^4–^ units, which display axially elongated 4 + 2 coordination (Figures 1c and S4), are connected into chains extending in *a*-crystallographic direction. The bond valence sum (BVS)^37^ analysis confirms the mixed +1/+2 valence state of silver (Table S1b). Furthermore, the monoclinic structural model of Ag^I^_2_Ag^II^F_4_ was also confirmed by the Rietveld refinement of neutron powder diffraction (NPD) (λ = 2.41 Å) data collected at 5 K on the mechanochemically synthesized Ag^I^_2_Ag^II^F_4_ sample (Figure 1d). Taking into account the difference in measurement temperatures, NPD-derived unit cell parameters are in excellent agreement with the synchrotron PXRD results, giving *a* = 3.5528(4) Å, *b* = 9.7931(12) Å, *c* = 5.9406(8) Å, β = 92.614(3)°, *V* = 206.48(8) Å^3^ (Table S2a). The Ag^I^–F bond distances [2.373(2)–2.834(3) Å] and three sets of Ag^II^–F lengths [2.096(2), 2.080(2) and 2.526(2) Å] (Table S2b) are also in good agreement with the structure determined by PXRD. Moreover, the amount of AgF impurity [10.6(1) wt %] detected in the NPD data is in accordance with the PXRD results [11.3(6) wt %] (Figure 1a).

The Rietveld refinement analysis of the synchrotron PXRD data measured on the sample synthesized by solid-state method also revealed a deviation from the expected weight ratio of 1.74:1 for AgF to AgF_2_ if the sample decomposed to the starting materials. Instead, it showed 22.9(1) wt % of AgF and only 0.9(5) wt % of AgF_2_ (Figure 1b). It can be assumed that this larger amount of AgF is the result of the sample decomposition in the capillary, as indicated by the loss of transparency of the glass. When the PXRD measurement was performed on a fresher sample, it showed a lower AgF content of 14.3(1) wt % (Figure S5). Nevertheless, mechanochemically synthesized samples of Ag_3_F_4_ were of superior purity than samples prepared by classical solid-state approach [88.7(2) and 89.4(1) vs 85.4(2) wt %].

**Figure 2:**
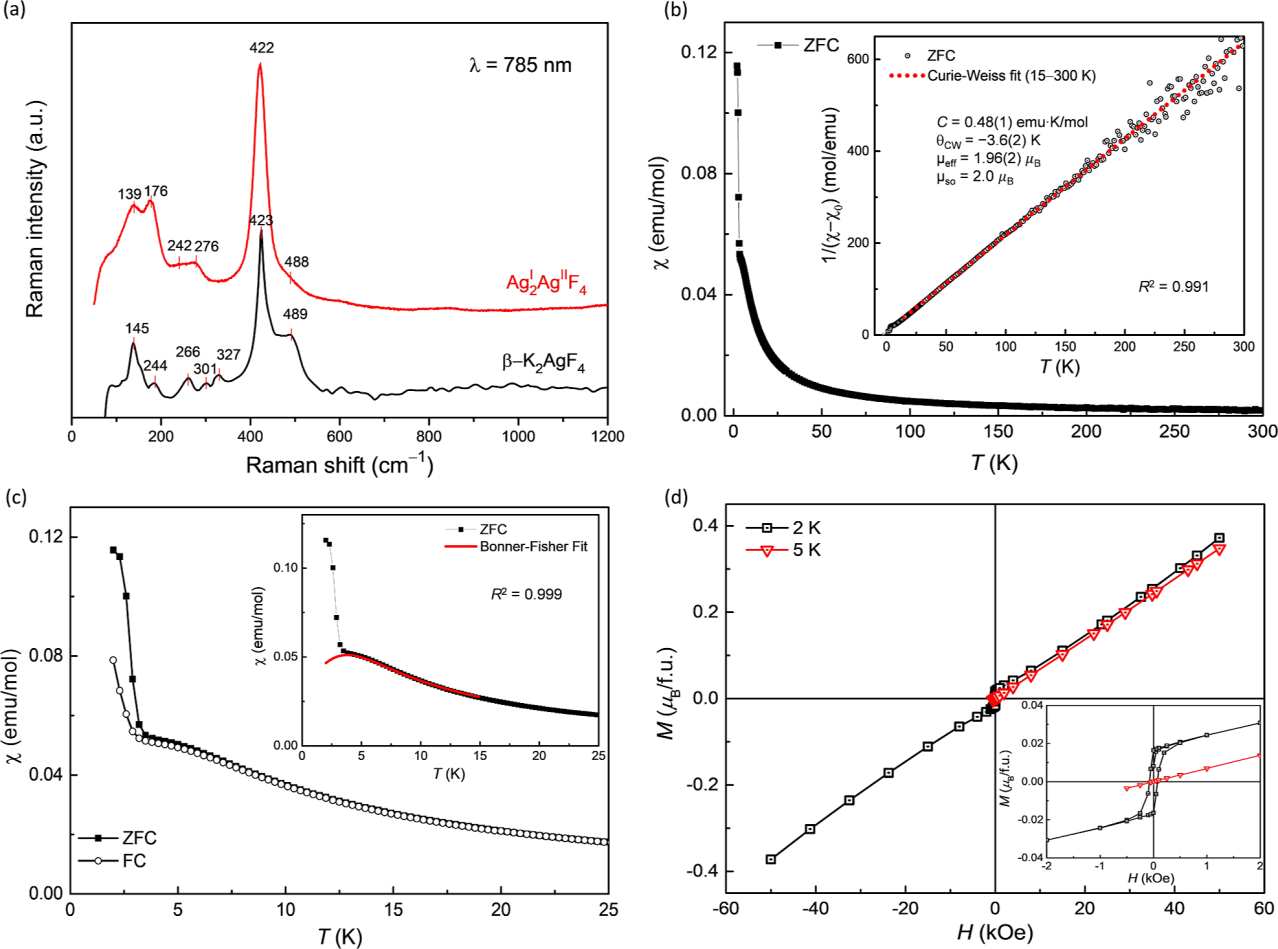
(a) Raman spectra of Ag^I^_2_Ag^II^F_4_ and β-K_2_AgF_4_ synthesized by ball milling. (b) Temperature-dependent magnetic susceptibility of mechanochemically prepared Ag^I^_2_Ag^II^F_4_ sample measured in a magnetic field of 1 kOe. The inset shows the Curie–Weiss fit to the inverse of magnetic susceptibility in the 15–300 K range. (c) Zero field cooled (ZFC) and field cooled (FC) data in the 2–25 K temperature region showing the upturn in the magnetic susceptibility characteristic of AFM, while the Bonner–Fisher fit for the 2–15 K region is shown in the inset. (d) Magnetization of Ag^I^_2_Ag^II^F_4_ as a function of magnetic field measured at 2 and 5 K. The hysteresis shown in the inset indicates a FM ground state.

An experimental indication of the existence of Ag^I^_2_Ag^II^F_4_ phase was also obtained from Raman measurements performed on AgF_2_ using a green excitation laser (λ = 532 nm). Longer illumination times led to changes in the Raman spectrum of AgF_2_; a decrease of the main band at 260 cm^–1^ and a significant increase of the minor band at 420 cm^–1^ (Figure S6) indicated a possible photochemical decomposition. Indeed, this spectrum is in excellent agreement with the Raman spectrum of the mechanochemically synthesized Ag_3_F_4_ sample. Both samples share the same spectral features, however the peaks in the mechanochemical sample are much broader. A similar observation on laser decomposition of AgF_2_ has been previously made but without the confirmation of the Ag_3_F_4_ phase formation.^38^ Since Ag^I^_2_Ag^II^F_4_ is isostructural to β-K_2_AgF_4_,^27^ similarities in their Raman spectra can be expected. To enable a direct comparison of Raman spectra measured under the same conditions, β-K_2_AgF_4_ was also synthesized mechanochemically (Figure S7). The successful formation of this phase further demonstrates the effectiveness of mechanochemistry for the synthesis of fluoridoargentates(II). The Raman spectrum of Ag^I^_2_Ag^II^F_4_ (Figure 2a) displays the most intense peak at 422 cm^–1^, which could be attributed to the symmetric vibrations of the [AgF_4_]^2–^ subunits and a shoulder at 488 cm^–1^. This is comparable to the peaks (423 and 489 cm^–1^) observed in the spectrum of β-K_2_AgF_4_, which is in good accordance with the literature reported values.^27^ Overall, the Raman spectra of both compounds display the same spectral features, confirming the structural similarities between the two phases. The results of the vibrational spectra thus also confirm that Ag^I^_2_Ag^II^F_4_ is isostructural to β-K_2_AgF_4_.

Given the structural similarities, the magnetic properties of Ag^I^_2_Ag^II^F_4_ can also be expected to resemble those of the β-K_2_AgF_4_ phase.^27^ The temperature-dependent magnetic susceptibility data (Figure 2b) shows that Ag^I^_2_Ag^II^F_4_ behaves like a paramagnet from room temperature down to about 5 K. At about 4 K, a broad peak can be observed, indicative of low-dimensional antiferromagnetic (AFM) properties, after which an abrupt increase in the magnetic susceptibility with lowering the temperature down to 3 K is observed, indicating a ferromagnetic (FM) transition. The value of the FM transition temperature was determined as 2.8(1) K from the maximum of the first derivative of the magnetic susceptibility, dχ/d*T*. The ball-milled and solid-state synthesized samples exhibit nearly identical magnetic responses (Figure S8), although the ball-milled sample displays a more pronounced broad maximum (see arrow).

The inverse magnetic susceptibility data was corrected for a constant diamagnetic contribution and fitted from 15 to 300 K with the Curie–Weiss law (Figure 2b, inset). The derived Curie constant of 0.48(1) emu·K/mol is in good agreement with the expected value for the *S* = 1/2 Ag^2+^ cations. A negative value of the Curie–Weiss temperature was determined, θ_CW_ = −3.6(2) K, suggesting that the dominant exchange is AFM. The 2–25 K region (Figure 2c) clearly shows the presence of a broad peak before the FM transition with a maximum at about 4 K, which is characteristic of a 1D AFM. A fit to the region around *T*_N_ (2–15 K) with a uniform AFM spin-1/2 chain Bonner–Fisher model^39^ (Figure 2c, inset) is excellent and the obtained parameters, *g* = 2.3 and |*J*_1D_|/*k*_B_ = 2.9 K are in good agreement with the values expected for a 1D AFM model, further supporting the 1D spin correlations above the *T*_N_ for the Ag^I^_2_Ag^II^F_4_ phase.

To gain a deeper insight into the magnetism of Ag^I^_2_Ag^II^F_4_, the *M*(*H*) curves were measured (Figure 2d). At 5 K, the magnetization shows a linear relationship with the applied magnetic field, without any remnant magnetization or coercivity. However, at 2 K, which is below the transition temperature, a pronounced hysteresis loop is observed, characterized by a remnant magnetization of approximately 0.0165 μ_B_/f.u. and a coercive field of *H*_c_ = 0.076 kOe. Even at the largest applied magnetic field of 50 kOe, the magnetization value remains modest at 0.07 μ_B_/f.u. and increases linearly with the strength of the magnetic field. The magnetization value falls significantly below the anticipated saturation magnetization of ≈1 μ_B_/f.u. for the *S* = 1/2 spin of the Ag^2+^ ion, which could be a result of a canted antiferromagnetism. A rough estimate of the canting angle (∼0.9°) in zero magnetic field could be obtained by comparing the measured remnant magnetization to the full magnetic moment of 1 μ_B_ per Ag^2+^ ion. The NPD measurements performed above (5 K) and below (1.5 K) the transition temperature [2.8(1) K] observed in the magnetic susceptibility data of the ball-milled Ag^I^_2_Ag^II^F_4_ sample show only one weak feature at around 16° 2θ in the 1.5 K data set (Figure S9, inset). This is not surprising, as weak magnetic Bragg peaks are in fact expected due to the low-dimensional magnetic structure and the small moment expected from the Ag^2+^ (*S* = 1/2). However, this sole peak was not sufficient to determine the magnetic structure of Ag^I^_2_Ag^II^F_4_.

### Ag^I^Ag^II^F_3_

**Figure 3:**
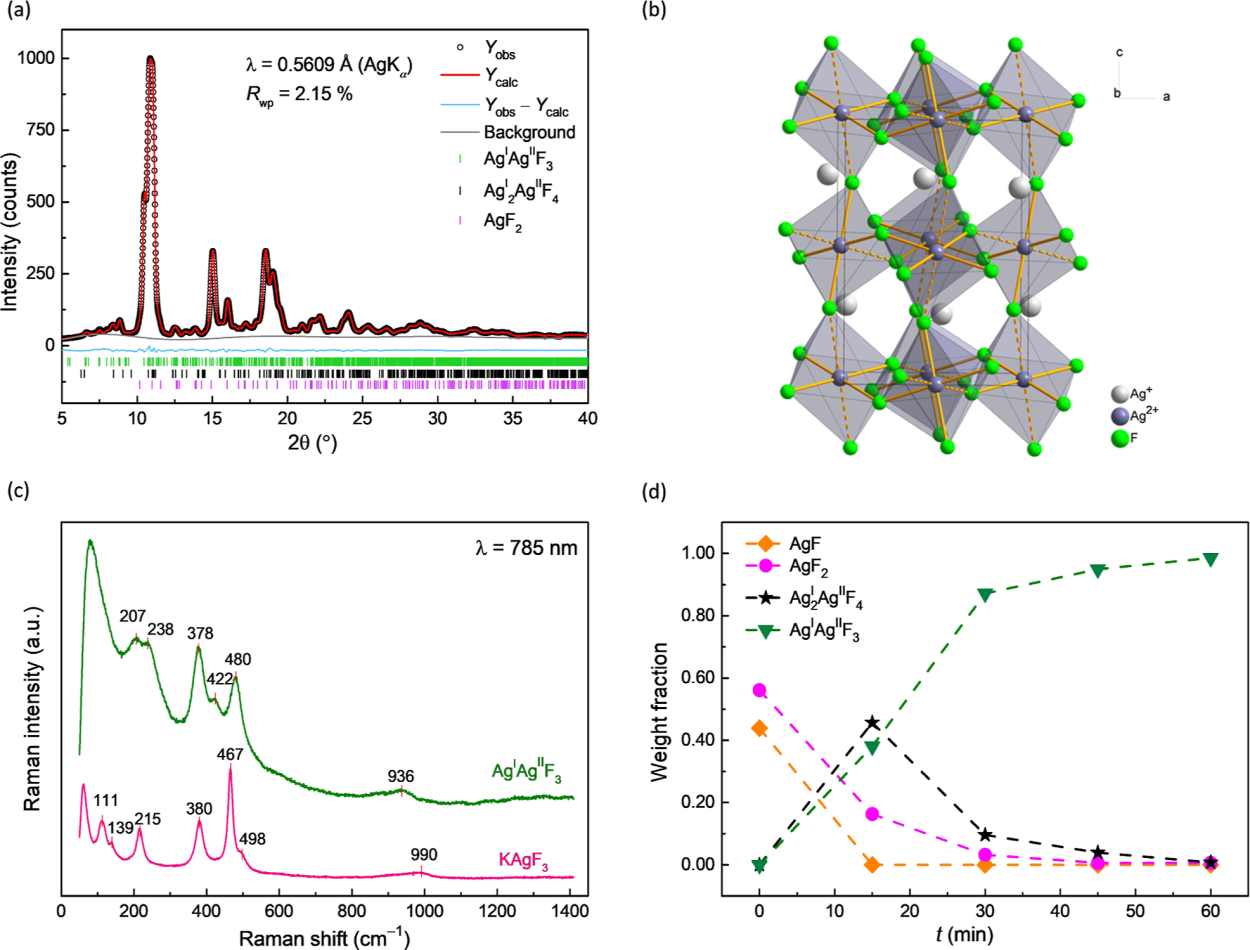
(a) Rietveld refinement of the laboratory PXRD data (Ag K_α_ radiation, λ = 0.5609 Å) of mechanochemically synthesized sample of Ag^I^Ag^II^F_3_, measured at room temperature. Quantitative analysis: Ag^I^Ag^II^F_3_: 95.6(1) wt %; Ag^I^_2_Ag^II^F_4_: 3.3(2) wt %; AgF_2_: 1.1(3) wt %. (b) Unit cell of the Ag^I^Ag^II^F_3_ (Ag_2_F_3_) crystal structure with apex-sharing [Ag^II^F_6/2_]^−^ distorted octahedral units with 4 + 2 coordination. (c) Raman spectrum of ball-milled Ag^I^Ag^II^F_3_. For comparison, the Raman spectrum of KAgF_3_ is also shown. (d) Weight fraction of phases in the ball-milled samples obtained from Rietveld refinement of the laboratory PXRD data as a function of milling time. The amorphous phase was not accounted in the refinement; the errors are smaller than the symbols in the graph.

The second stoichiometry that was thoroughly investigated in this study was a 1:1 AgF–AgF_2_ molar ratio. The experimental details are described in the Supporting Information. Note that cooling the milling jars with liquid nitrogen was essential in stabilizing this new phase and minimizing its decomposition to Ag^I^_2_Ag^II^F_4_ and AgF_2_. The laboratory PXRD data on the light-brown powder (Figure 3a) confirmed the successful mechanochemical formation of a new phase Ag^I^Ag^II^F_3_ [95.6(1) wt %] with minor impurities Ag_3_F_4_ [3.3(2) wt %] and AgF_2_ [1.1(3) wt %]. Solid state synthesis was also performed at 300 °C and the synchrotron PXRD data collected on this sample was used to index the peaks of the new Ag^I^Ag^II^F_3_ phase (Figure S10). A triclinic *P*1̅ space group was found, with a unit cell similar to that of AgCuF_3_,^40^ resembling a distorted MAgF_3_ structure (M = K, Rb and Cs).^41^ A Rietveld refinement performed using the triclinic *P*1̅ space group resulted in excellent fit and the following unit cell parameters: *a* = 5.9577(1) Å, *b* = 5.8217(1) Å, *c* = 8.5467(2) Å, α = 91.565(2), β = 90.644(2), γ = 85.977(1)°, *V* = 295.582(6) Å^3^, *Z* = 4 (Figure S11, Table S3a). Thus, Ag^I^Ag^II^F_3_ is isostructural to AgCuF_3_,^40^ and adopts the NaCuF_3_ structure type (Figure 3b).^42^ In the crystal structure of Ag_2_F_3_, there are two nonequivalent silver(I) atoms, both coordinated with seven fluorine atoms [2.341(16)–3.072(20) Å]. Moreover, four nonequivalent silver(II) atoms are all coordinated with six fluorine atoms, forming a [AgF_6_]^4–^ octahedra with four short [2.066(11)–2.161(13) Å] and two long [2.403(11)–2.441(16) Å] Ag^II^–F distances (Table S3b, Figures S12 and S13). The octahedra thus exhibit an axial elongation (4 + 2) and could therefore be Jahn–Teller active. The corner-sharing [AgF_6/2_]^−^ units are connected into a distorted perovskite structure, resembling a structural arrangement of the KAgF_3_ compound (Figure S12).^41,43^ The BVS analysis^37^ (Table S3b) confirms the valence state of the silver(I) and silver(II) atoms and provides further support for the correctness of the determined crystal structure.

The Raman spectrum of Ag^I^Ag^II^F_3_ was measured and compared to the literature reported one for KAgF_3_.^44^ For a detailed comparison of Raman spectra measured under the same conditions, KAgF_3_ was also synthesized. Raman spectra of Ag_2_F_3_ and KAgF_3_ exhibit very similar features (Figure 3c) with the most intense peaks observed at 480, 378 and 467, 380 cm^–1^, respectively.

It is noteworthy, that the most intense Raman active mode of Ag^I^_2_Ag^II^F_4_ located at 422 cm^–1^ is also present in the Raman spectrum of Ag^I^Ag^II^F_3_, albeit with a very weak signal due to the low content. The presence of the Ag_3_F_4_ phase could be explained by the observation that the mechanochemically synthesized Ag^I^Ag^II^F_3_ sample stored in the glovebox at room temperature decomposes into AgF_2_ and Ag^I^_2_Ag^II^F_4_ over time (Figure S14).

To gain a deeper insight into the reaction mechanism of Ag_3_F_4_ formation, time-dependent milling experiments with sampling every 15 min were performed (Figure S15). The analysis reveals that after 15 min of milling, the majority of the AgF was consumed and the milled sample consisted mainly of AgF_2_, Ag^I^Ag^II^F_3_ and Ag^I^_2_Ag^II^F_4_ (Figure 3d). After 60 min of milling, the sample contained Ag^I^Ag^II^F_3_ with traces of the Ag^I^_2_Ag^II^F_4_ phase. It can thus be assumed that Ag^I^Ag^II^F_3_ is formed from the reaction of the intermediate Ag^I^_2_Ag^II^F_4_ phase with unreacted AgF_2_ (eq 1)
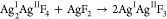
1

**Figure 4:**
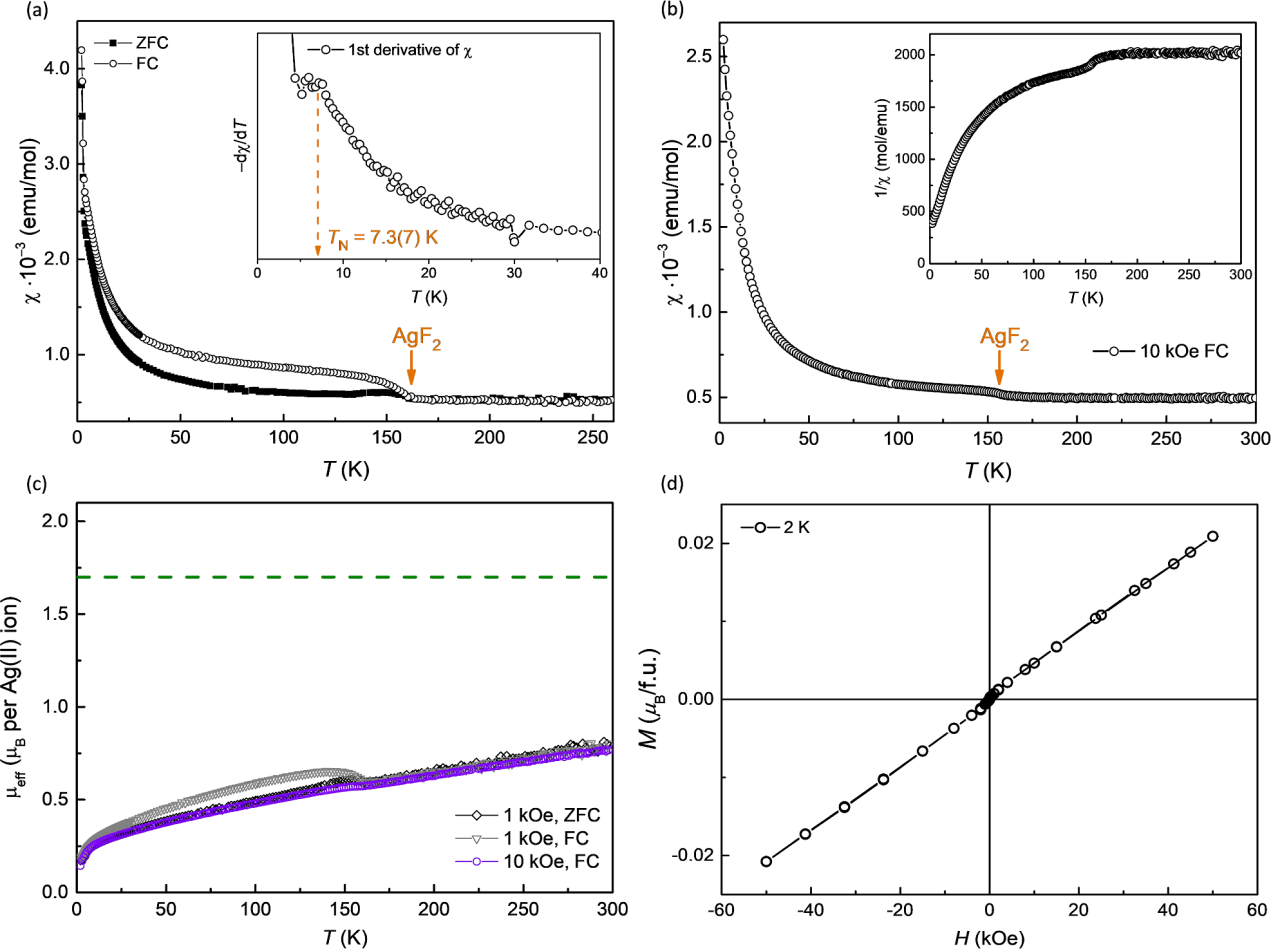
Temperature-dependent magnetic susceptibility, χ, of Ag^I^Ag^II^F_3_, measured in a magnetic field of (a) 1 and (b) 10 kOe. The inset in (a) shows the first derivative indicating a deviation from the Curie–Weiss law at 7.3(7) K. The inset in (b) shows the inverse of the magnetic susceptibility (FC) measured at 10 kOe. (c) Plot of the effective magnetic moment, μ_eff_, as a function of temperature for FC and ZFC curves at 1 kOe and for the FC curve measured at 10 kOe. (d) Magnetization of Ag^I^Ag^II^F_3_ as a function of magnetic field, measured at 2 K.

Furthermore, the magnetic susceptibility data measured on several mechanochemically synthesized samples reveals intriguing features that provide valuable insights into the magnetic properties of Ag^I^Ag^II^F_3_ (Figures 4a and S16).

A weak AgF_2_ impurity signal is observed at approximately 164 K and quantified from the χ·*T* data to about 0.8(3) wt % AgF_2_, which is in good agreement with the value of 1.1(3) wt % obtained from the PXRD analysis (Figure 3a). The presence of AgF_2_ is expected due to either the 10 mol % excess of AgF_2_ used in the synthesis or a decomposition of the Ag_2_F_3_ phase into Ag_3_F_4_ phase and AgF_2_. A weak FM signal corresponding to the Ag_3_F_4_ phase [3.3(2) wt %] is also observed. It should be noted that when the magnetization is measured on the Ag^I^Ag^II^F_3_ sample synthesized mechanochemically with a stoichiometric 1:1 AgF–AgF_2_ molar ratio, a smaller amount of AgF_2_ is present, but a larger amount of the Ag_3_F_4_ phase is observed as a secondary phase, which is minimized when an excess of AgF_2_ is used in the synthesis.

The first derivative of magnetic susceptibility indicates the temperature of 7.3(7) K at which the deviation from the Curie–Weiss law can be observed (Figure 4a, inset). The magnetic susceptibility was also measured in a ten times larger magnetic field of 10 kOe to minimize the influence of the FM signal of AgF_2_ and other possible FM impurities (Figure 4b). The high-temperature range (200–300 K) of the 10 kOe measurement shows a persistent, continuous decrease in susceptibility with decreasing temperature, indicative of AFM interactions persisting even at elevated temperatures. The observed behavior resembles KAgF_3_, a 1D antiferromagnet.^44^ This hypothesis is also supported by the fact that the effective magnetic moment at 300 K is well below the theoretically expected value of 1.7 μ_B_, reaching only 0.8 μ_B_ (Figure 4c). The effective magnetic moment also decreases with decreasing temperature and reaches a value of approximately 0.15 μ_B_, indicating AFM interactions are predominant throughout the whole measured temperature range. In addition, the magnetization measurements (Figure 4d) show that the magnetic moments do not attain saturation even under the influence of high magnetic fields, a characteristic that strongly suggests the presence of AFM interactions.

## Conclusions

In this study, mechanochemistry was employed as a novel synthetic method for fluoridoargentates(II) and provided the first experimental evidence for two binary mixed-valent silver(I,II) fluorides, Ag^I^_2_Ag^II^F_4_ (Ag_3_F_4_) and Ag^I^Ag^II^F_3_ (Ag_2_F_3_). The Ag^I^_2_Ag^II^F_4_ phase is stable at room temperature, whereas the Ag^I^Ag^II^F_3_ phase appears to be metastable and required milling at liquid nitrogen temperatures. Synchrotron PXRD, NPD, and Raman spectroscopy studies confirmed the Na_2_CuF_4_-type structure of Ag^I^_2_Ag^II^F_4_, which is similar to that of β-K_2_AgF_4_. This novel compound shows canted antiferromagnetism below 2.8(1) K. On the other side, the triclinic perovskite Ag^I^Ag^II^F_3_ phase, isostructural to AgCuF_3_, shares structural features with KAgF_3_, and shows characteristics of a one-dimensional antiferromagnet. This work thus demonstrates the effectiveness of mechanochemical approach for the synthesis of fluoridoargentates(II), as it provides samples of Ag_3_F_4_, Ag_2_F_3_, and β-K_2_AgF_4_ with superior purity in comparison to the conventional solid-state thermal route. Moreover, mechanochemistry enables a low-temperature synthesis, addressing the challenges posed by Ag^2+^ reactivity and thermal instability of AgF_2_. This approach not only facilitates the discovery of new silver(II) compounds but also enables the expansion of this relatively underexplored area of chemistry.

## Supporting Information Available

The experimental procedures, description of the characterization techniques, and results of analyses (PDF)

## Dedication

This work is dedicated to the memory of Professor Boris Žemva, whose passion for fluorine chemistry ignited our pursuit in this field.

## References

[ref1] Broholm C.; Cava R. J.; Kivelson S. A.; Nocera D. G.; Norman M. R.; Senthil T. Quantum spin liquids. Science 2020, 367 (6475), eaay0668 10.1126/science.aay0668. 31949052

[ref2] Anderson P. W. The Resonating Valence Bond State in La_2_CuO_4_ and Superconductivity. Science 1987, 235 (4793), 1196–1198. 10.1126/science.235.4793.1196. 17818979

[ref3] Grochala W.; Hoffmann R. Real and Hypothetical Intermediate-Valence Ag^II^/Ag^III^ and Ag^II^/Ag^I^ Fluoride Systems as Potential Superconductors. Angew. Chem., Int. Ed. 2001, 40 (15), 2742–2781. 10.1002/1521-3773(20010803)40:15<2742::AID-ANIE2742>3.0.CO;2-X. 29711991

[ref4] Grochala W. The theory-driven quest for a novel family of superconductors: fluorides. J. Mater. Chem. 2009, 19 (38), 6949–6968. 10.1039/b904204k.

[ref5] Žemva B.; Hagiwara R.; Casteel W. J.; Lutar K.; Jesih A.; Bartlett N. Spontaneous oxidation of xenon to Xe(II) by cationic Ag(II) in anhydrous hydrogen fluoride solutions. J. Am. Chem. Soc. 1990, 112 (12), 4846–4849. 10.1021/ja00168a032.

[ref6] Žemva B. Protonic superacid anhydrous hydrogen fluoride as a solvent in the chemistry of high oxidation states. C. R. Acad. Sci., Ser. IIc: Chim. 1998, 1 (3), 151–156. 10.1016/S1387-1609(99)80073-5.

[ref7] Grochala W. Silverland: the Realm of Compounds of Divalent Silver—and Why They are Interesting. J. Supercond. Novel Magn. 2018, 31, 737–752. 10.1007/s10948-017-4326-8.

[ref8] Gawraczyński J.; Kurzydłowski D.; Ewings R. A.; Bandaru S.; Gadomski W.; Mazej Z.; Ruani G.; Bergenti I.; Jaroń T.; Ozarowski A.; Hill S.; Leszczyński P. J.; Tokár K.; Derzsi M.; Barone P.; Wohlfeld K.; Lorenzana J.; Grochala W. Silver route to cuprate analogs. Proc. Natl. Acad. Sci. U.S.A. 2019, 116 (5), 1495–1500. 10.1073/pnas.1812857116. 30651308 PMC6358696

[ref9] Grochala W.; Egdell R. G.; Edwards P. P.; Mazej Z.; Žemva B. On the Covalency of Silver–Fluorine Bonds in Compounds of Silver(I), Silver(II) and Silver(III). ChemPhysChem 2003, 4 (9), 997–1001. 10.1002/cphc.200300777. 14562447

[ref10] Bachar N.; Koteras K.; Gawraczynski J.; Trzciński W.; Paszula J.; Piombo R.; Barone P.; Mazej Z.; Ghiringhelli G.; Nag A.; Zhou K.-J.; Lorenzana J.; Van Der Marel D.; Grochala W. Charge-Transfer and *dd* excitations in AgF_2_. Phys. Rev. Res. 2022, 4 (2), 023108 10.1103/PhysRevResearch.4.023108.

[ref11] Hettich A. Über die Natur von Silbersubfluorid. Z. Anorg. Allg. Chem. 1927, 167 (1), 67–74. 10.1002/zaac.19271670106.

[ref12] Terrey H.; Diamond H. The Crystal Structure of Silver Subfluoride. J. Chem. Soc. 1928, 2820–2824. 10.1039/JR9280002820.

[ref13] Andres K.; Kuebler N. A.; Robin M. B. Superconductivity in Ag_2_F. J. Phys. Chem. Solids 1966, 27 (11–12), 1747–1748. 10.1016/0022-3697(66)90104-1.

[ref14] Ott H. Die Strukturen von MnO, MnS, AgF, NiS, SnJ_4_, SrCl_2_, BaF_2_; Präzisionsmessungen einiger Alkalihalogenide. Z. Kristallogr. 1926, 63 (1–6), 222–230. 10.1524/zkri.1926.63.1.222.

[ref15] Lozinšek M.; Belak Vivod M.; Dragomir M. Crystal structure reinvestigation of silver(I) fluoride, AgF. IUCrData 2023, 8, x230018 10.1107/S2414314623000184. 36794053 PMC9912324

[ref16] Ebert M. S.; Rodowskas E. L.; Frazer J. C. W. Higher valence states of silver. J. Am. Chem. Soc. 1933, 55 (7), 3056–3057. 10.1021/ja01334a514.

[ref17] Ruff O.; Giese M. Die Fluorierung des Silbers und Kupfers. Z. Anorg. Allg. Chem. 1934, 219 (2), 143–148. 10.1002/zaac.19342190206.

[ref18] Jesih A.; Lutar K.; Žemva B.; Bachmann B.; Becker S.; Müller B. G.; Hoppe R. Einkristalluntersuchungen an AgF_2_. Z. Anorg. Allg. Chem. 1990, 588 (1), 77–83. 10.1002/zaac.19905880110.

[ref19] Bougon R.; Bui Huy T.; Lance M.; Abazli H. Synthesis and Properties of Silver Trifluoride, AgF_3_. Inorg. Chem. 1984, 23 (22), 3667–3668. 10.1021/ic00190a049.

[ref20] Žemva B.; Lutar K.; Jesih A.; Casteel W. J.; Wilkinson A. P.; Cox D. E.; von Dreele R. B.; Borrmann H.; Bartlett N. Silver trifluoride: preparation, crystal structure, some properties, and comparison with AuF_3_. J. Am. Chem. Soc. 1991, 113 (11), 4192–4198. 10.1021/ja00011a021.

[ref21] Fischer R.; Müller B. G. Die Kristallstruktur von Ag^II^F[Ag^III^F_4_]. Z. Anorg. Allg. Chem. 2002, 628 (12), 2592–2596. 10.1002/1521-3749(200212)628:12<2592::AID-ZAAC2592>3.0.CO;2-O.

[ref22] Graudejus O.; Wilkinson A. P.; Bartlett N. Structural Features of Ag[AuF_4_] and Ag[AuF_6_] and the Structural Relationship of Ag[AgF_4_]_2_ and Au[AuF_4_]_2_ to Ag[AuF_4_]_2_. Inorg. Chem. 2000, 39 (7), 1545–1548. 10.1021/ic991178t. 12526462

[ref23] Grochala W. On possible existence of pseudobinary mixed valence fluorides of Ag(I)/Ag(II): a DFT study. J. Mol. Model. 2011, 17, 2237–2248. 10.1007/s00894-010-0949-4. 21258832

[ref24] Kurzydłowski D.; Derzsi M.; Zurek E.; Grochala W. Fluorides of silver under large compression. Chem.—Eur. J. 2021, 27 (17), 5536–5545. 10.1002/chem.202100028. 33471421

[ref25] Rybin N.; Chepkasov I.; Novoselov D. Y.; Anisimov V. I.; Oganov A. R. Prediction of Stable Silver Fluorides. J. Phys. Chem. C 2022, 126 (35), 15057–15063. 10.1021/acs.jpcc.2c04785.

[ref26] Babel D. Untersuchungen an ternären Fluoriden. III. Die Struktur des Na_2_CuF_4_. Z. Anorg. Allg. Chem. 1965, 336 (3–4), 200–206. 10.1002/zaac.19653360310.

[ref27] Kurzydłowski D.; Derzsi M.; Budzianowski A.; Jagličić Z.; Koźmiński W.; Mazej Z.; Grochala W. Polymorphism of Fluoroargentates(II): Facile Collapse of a Layered Network of α-K_2_AgF_4_ Due to the Insufficient Size of the Potassium Cation. Eur. J. Inorg. Chem. 2010, (19), 2919–2925. 10.1002/ejic.201000124.

[ref28] Baláž P.; Achimovičová M.; Baláž M.; Billik P.; Cherkezova-Zheleva Z.; Criado J. M.; Delogu F.; Dutková E.; Gaffet E.; Gotor F. J.; Kumar R.; Mitov I.; Rojac T.; Senna M.; Streletskii A.; Wieczorek-Ciurowa K. Hallmarks of mechanochemistry: from nanoparticles to technology. Chem. Soc. Rev. 2013, 42 (18), 7571–7637. 10.1039/C3CS35468G. 23558752

[ref29] Tan D.; Garcia F. Main group mechanochemistry: from curiosity to established protocols. Chem. Soc. Rev. 2019, 48 (8), 2274–2292. 10.1039/C7CS00813A. 30806391

[ref30] Friščić T.; Mottillo C.; Titi H. M. Mechanochemistry for Synthesis. Angew. Chem., Int. Ed. 2020, 59 (3), 1018–1029. 10.1002/anie.201906755. 31294885

[ref31] Martinez V.; Stolar T.; Karadeniz B.; Brekalo I.; Užarević K. Advancing mechanochemical synthesis by combining milling with different energy sources. Nat. Rev. Chem 2023, 7, 51–65. 10.1038/s41570-022-00442-1. 37117822

[ref32] Šepelák V.; Düvel A.; Wilkening M.; Becker K. D.; Heitjans P. Mechanochemical reactions and syntheses of oxides. Chem. Soc. Rev. 2013, 42 (18), 7507–7520. 10.1039/c2cs35462d. 23364473

[ref33] Preishuber-Pflügl F.; Wilkening M. Mechanochemically synthesized fluorides: local structures and ion transport. Dalton Trans. 2016, 45 (21), 8675–8687. 10.1039/C6DT00944A. 27172256

[ref34] Ruprecht B.; Wilkening M.; Feldhoff A.; Steuernagel S.; Heitjans P. High anion conductivity in a ternary non-equilibrium phase of BaF_2_ and CaF_2_ with mixed cations. Phys. Chem. Chem. Phys. 2009, 11 (17), 3071–3081. 10.1039/b901293a. 19370201

[ref35] Kurzydłowski D.; Jaroń T.; Ozarowski A.; Hill S.; Jagličić Z.; Filinchuk Y.; Mazej Z.; Grochala W. Local and Cooperative Jahn–Teller Effect and Resultant Magnetic Properties of M_2_AgF_4_ (M = Na–Cs) Phases. Inorg. Chem. 2016, 55 (21), 11479–11489. 10.1021/acs.inorgchem.6b02037. 27753481

[ref36] Lozinšek M.; Goreshnik G.; Žemva B. Silver(I) Tetrafluoridooxidovanadate(V) – Ag[VOF_4_]. Acta Chim. Slov. 2014, 61 (3), 542–547. 25286209

[ref37] Brown I. D. Recent Developments in the Methods and Applications of the Bond Valence Model. Chem. Rev. 2009, 109 (12), 6858–6919. 10.1021/cr900053k. 19728716 PMC2791485

[ref38] Gawraczyński J. Optical Spectroscopy of Selected Divalent Silver Compounds. Ph.D. Dissertation, University of Warsaw, 2019.

[ref39] Bonner J. C.; Fisher M. E. Linear Magnetic Chains with Anisotropic Coupling. Phys. Rev. 1964, 135 (3A), A640–A658. 10.1103/PhysRev.135.A640.

[ref40] Tong J.; Lee C.; Whangbo M. H.; Kremer R. K.; Simon A.; Köhler J. Cooperative Jahn–Teller distortion leading to the spin-1/2 uniform antiferromagnetic chains in triclinic perovskites AgCuF_3_ and NaCuF_3_. Solid State Sci. 2010, 12 (5), 680–684. 10.1016/j.solidstatesciences.2009.02.028.

[ref41] Odenthal R. H.; Hoppe R. Fluoroargentate(II) der Alkalimetalle. Monatsh. Chem. 1971, 102, 1340–1350. 10.1007/BF00917190.

[ref42] Kaiser V.; Otto M.; Binder F.; Babel D. Jahn-Teller-Effekt und Kristallstruktur-Verzerrung bei den Kupfer-Fluorperowskiten NaCuF_3_ und RbCuF_3_. Z. Anorg. Allg. Chem. 1990, 585 (1), 93–104. 10.1002/zaac.19905850112.

[ref43] Mazej Z.; Goreshnik E.; Jagličić Z.; Gaweł B.; Łasocha W.; Grzybowska D.; Jaroń T.; Kurzydłowski D.; Malinowski P.; Koźminski W.; Szydłowska J.; Leszczyński P.; Grochala W. KAgF_3_, K_2_AgF_4_ and K_3_Ag_2_F_7_: important steps towards a layered antiferromagnetic fluoroargentate(II). CrystEngComm 2009, 11 (8), 1702–1710. 10.1039/B902161B.

[ref44] Kurzydłowski D.; Mazej Z.; Jagličić Z.; Filinchuk Y.; Grochala W. Structural transition and unusually strong antiferromagnetic superexchange coupling in perovskite KAgF_3_. Chem. Commun. 2013, 49 (56), 6262–6264. 10.1039/c3cc41521j. 23598949

