# Supplementary material for: Mechanochemical Synthesis and Magnetic Properties of the Mixed-Valent Binary Silver(I,II) Fluorides, AgI2AgIIF4 and AgIAgIIF3
Source: J Am Chem Soc. 2024 Oct 24;146(44):30510–7. doi: 10.1021/jacs.4c11772 (PMC11544617; doi:10.1021/jacs.4c11772)
Supplement: Supplementary file 1 — ja4c11772_si_001.pdf [file ja4c11772_si_001.pdf]

Supporting Information

Mechanochemical Synthesis and Magnetic Properties of the Mixed-Valent Binary Silver(I,II) Fluorides, $Ag^{I}_{2}Ag^{II}F_4$ and $Ag^{I}Ag^{II}F_3$

Matic Belak Vivod,[a,b] Zvonko Jagličić,[c,d] Graham King,[e] Thomas C. Hansen,[f] Matic Lozinšek,[a,b] and Mirela Dragomir[a,b]*

[a] *Jožef Stefan Institute, Jamova cesta 39, 1000 Ljubljana, Slovenia*

[b] *Jožef Stefan International Postgraduate School, Jamova cesta 39, 1000 Ljubljana, Slovenia*

[c] *Institute of Mathematics, Physics and Mechanics, 1000 Ljubljana, Slovenia*

[d] *Faculty of Civil and Geodetic Engineering, University of Ljubljana, Jamova cesta 2, 1000 Ljubljana, Slovenia*

[e] *Canadian Light Source, 44 Innovation Blvd, Saskatoon, SK S7N 2V3, Canada*

[f] *Institut Laue-Langevin, 38042 Grenoble Cedex 9, France*

*Corresponding author e-mail: mirela.dragomir@ijs.si

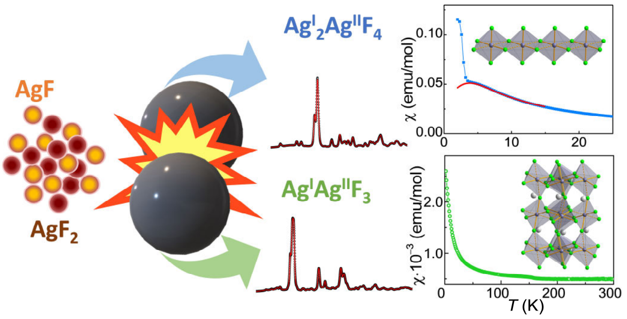

CONTENTS

SECTION A Synthetic Procedures 3

SECTION B Characterization 4

SECTION C Results – $Ag^{I}_{2}Ag^{II}F_4$ 5

SECTION D Results – $Ag^{I}Ag^{II}F_3$ 12

SECTION E References 18

SECTION A Synthetic Procedures

Caution!

Toxic hydrogen fluoride (HF) is produced upon the hydrolysis of metal fluorides. Silver difluoride is a potent oxidizing agent. To ensure safe handling of these compounds, the use of appropriate protective gear is essential.

Materials

$Ag^{I}_2Ag^{II}F_4$, $Ag^{I}Ag^{II}F_3$, K_2AgF_4 and $KAgF_3$ were synthesized from commercially available AgF_2 (98% Sigma-Aldrich or 99% Fluorochem), AgF (99% Thermo Scientific or 99.9% Sigma-Aldrich) and KF (99% Fluorochem), which were used as received. Handling of all chemicals was performed in an argon-filled glovebox (M. Braun, Germany) with water and oxygen levels below 0.1 ppm.

The synthesized samples were handled in the glovebox under inert conditions at all times.

Mechanochemical synthesis

For the mechanochemical synthesis of $Ag^{I}_2Ag^{II}F_4$, approximately 1 g of a 2:1 or 2:1.1 AgF–AgF_2 molar mixture was hand-homogenized in an agate mortar for 15 minutes. The homogenized mixture was then transferred into a 10 ml tungsten carbide milling jar equipped with a PTFE gasket along with a tungsten carbide milling ball with a diameter of 15 mm. The ball-to-reagent ratio (BRR) was 22. The milling was carried out using a Retsch MM 400 mixer mill. The powder mixture was milled for a total of 30 minutes divided into 2 cycles, with each cycle consisting of 15 minutes milling followed by 15-minutes of cooling at room temperature. The same reaction conditions were used for the synthesis of β-K_2AgF_4, using a 10 mol% excess of AgF_2 and a total milling time of 90 minutes (6 cycles).

For the mechanochemical synthesis of $Ag^{I}Ag^{II}F_3$, 1 g of hand-homogenized powder with a 1:1 or 1:1.1 AgF–AgF_2 molar ratio was placed into a 10 ml stainless steel milling jar equipped with a PTFE gasket, along with a stainless-steel milling ball with diameter of 15 mm (BRR was 12). The powder mixture was milled for a total of 60 minutes in 4 cycles. The jars were pre-cooled in liquid nitrogen for about 5 minutes before milling, and further cooled by immersion in liquid nitrogen between milling cycles.

Solid-state synthesis

In a typical solid-state synthesis of $Ag^{I}_2Ag^{II}F_4$ and $Ag^{I}Ag^{II}F_3$, a 1:1.1 or 2:1.1 AgF–AgF_2 molar mixture (about 300 mg) was hand-homogenized in an agate mortar and then placed in a platinum crucible which was enclosed in a nickel reactor in Ar atmosphere and heated to 300 °C for 3 days. After the reaction was finalized, the sample was naturally cooled to room temperature. It was observed that if the reaction was carried out at higher temperatures the sample decomposed. The $KAgF_3$ was prepared according to a previously reported literature method.[S1]

SECTION B Characterization

Powder X-ray diffraction

The homogenized samples were transferred to dried quartz capillaries (0.3 mm in diameter, Hilgenberg) and flame-sealed using a hydrogen–oxygen micro torch. Laboratory powder X-ray diffraction patterns were measured on a Rigaku OD XtaLAB Synergy-S, Dualflex diffractometer, equipped with an Eiger2 R CdTe 1M hybrid pixel array detector, using microfocus Ag Kα radiation (λ = 0.56087 Å, 65 kV, 0.67 mA). The data was collected in transmission mode at room temperature and at 100 K, with a detector distance of 90 mm. Phases were identified using *X'Pert HighScore Plus*[S2] with the *PDF-4+* database. The powder patterns were processed and extracted by *CrysAlis^Pro^* software.[S3]

Synchrotron powder X-ray diffraction patterns were measured in quartz capillaries with 0.3 mm diameter (Hilgenberg) at the Brockhouse High Energy Wiggler Beamline of the Canadian Light Source (CLS) using λ = 0.3497 Å radiation and a Varex area detector. Indexing and Rietveld refinements analyses were performed using *GSAS-II* software.[S4]

Neutron powder diffraction

Approximately 1 g of mechanochemically synthesized $Ag^{I}_2Ag^{II}F_4$ was loaded into a vanadium can inside the glovebox. The sample was measured at the high intensity powder diffractometer D20 at the Institute Laue-Langevin (ILL), France, employing a wavelength of λ = 2.41 Å from 2 K to 5 K.[S5]

Raman spectroscopy

Raman spectra were measured in quartz capillaries at room temperature using a Bruker Senterra II confocal Raman microscope with a 785 nm emission line and an output power of 1 mW. The spectra were measured in the range from 50 to 1410 cm^{-1} with a resolution of 1.5 cm^{-1}. Additionally, a green emission line (532 nm, 12.5 mW) was used to study the photochemical decomposition of AgF_2.

Magnetization

Magnetic susceptibility measurements were performed using a Quantum Design MPMS-XL-5 SQUID magnetometer. Temperature-dependent magnetic susceptibility (χ) was measured from 2 K to 300 K in constant magnetic fields of 1 kOe and 10 kOe. Isothermal magnetization was measured at temperatures of 2 K and 5 K between −50 and +50 kOe. The data were corrected for the experimentally determined contribution of the quartz capillary and the diamagnetic response of the compound due to closed atomic shells obtained from Pascal's tables.[S6]

SECTION D Results – $Ag^{I}_{2}Ag^{II}F_4$

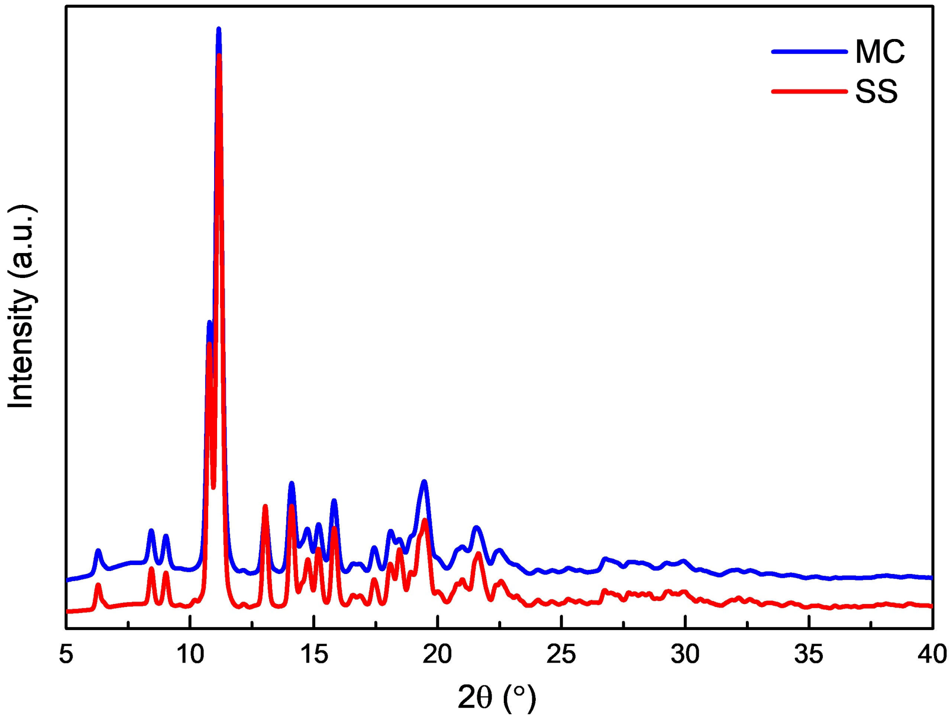

Figure S1. Laboratory PXRD data of $Ag^{I}_{2}Ag^{II}F_4$ prepared by both mechanochemistry (MC) and solid-state synthesis (SS), measured at room temperature with Ag Kα radiation (λ = 0.5609 Å).

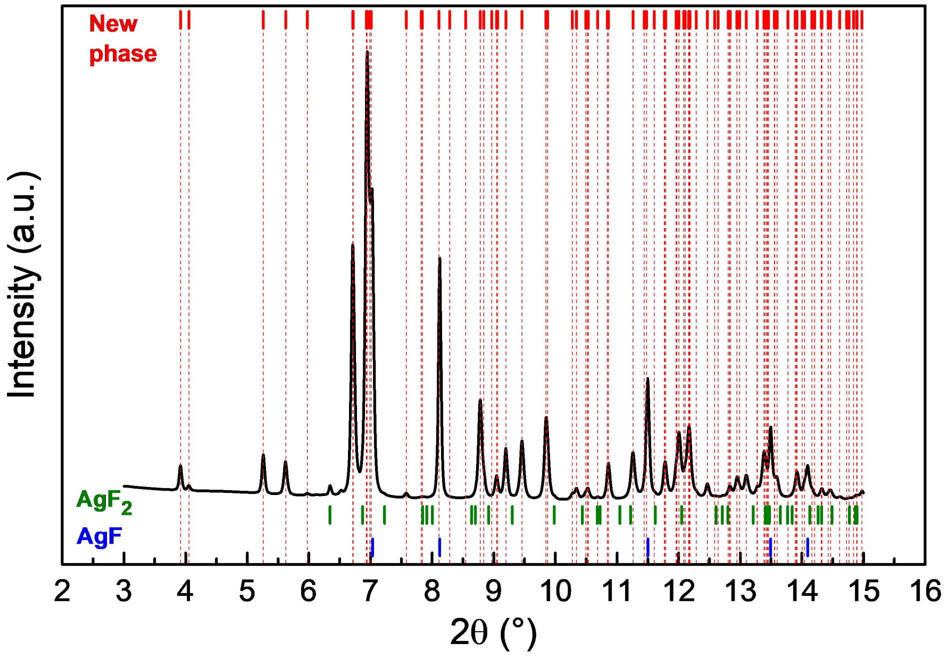

Figure S2. Unit cell indexing ($P2_1/c$ space group) of the synchrotron PXRD data (λ = 0.3497 Å) measured on $Ag^{I}_{2}Ag^{II}F_4$ sample prepared by the solid-state synthesis. For clarity, only a selected range is shown.

Table S1a. Crystallographic parameters of $Ag^{I}_2Ag^{II}F_4$ obtained by the Rietveld refinement of room-temperature synchrotron data (λ = 0.3497 Å).

$Ag^{I}_2Ag^{II}F_4$	
**Space group**	$P2_1/c$ (no. 14)
**Chemical formula weight** (g/mol)	399.6
Z	2
a*, *b*, *c (Å)	3.56910(8), 9.88659(15), 5.9829(2)
β (°)	92.8321(15)
V (Å^3)	210.857(4)
**Ag1** (*x*, *y*, *z*)<br>U_{iso}<br>**Oxidation state**	0, 0, 0<br>0.0110(4)<br>+2
**Ag2** (*x*, *y*, *z*)<br>U_{iso}<br>**Oxidation state**	0.5122(3), 0.17859(15), 0.43044(15)<br>0.0162(3)<br>+1
**F1** (*x*, *y*, *z*)<br>U_{iso}	0.5568(18), 0.4522(8), 0.2668(11)<br>0.053(3)
**F2** (*x*, *y*, *z*)<br>U_{iso}	0.0155(18), 0.1971(8), 0.1391(10)<br>0.0212(17)
GOF	1.61
R_{wp}	0.0108

Table S1b. Interatomic distances and bond valence sum analysis[S7] for silver atoms in $Ag^{I}_2Ag^{II}F_4$. The following bond valence parameters were employed: b = 0.37 Å; R_0 = 1.80 Å (Ag^{I}–F) and 1.79 Å (Ag^{II}–F).[S8,S9]

**Interatomic distances** (Å)			
**Ag1–F1** (× 2)	2.110(6)	**Ag2–F1**	2.391(7)
**Ag1–F2** (× 2)	2.119(7)	**Ag2–F2**	2.431(7)
**Ag1–F1** (× 2)	2.529(7)	**Ag2–F2**	2.464(7)
		**Ag2–F2**	2.536(7)
		**Ag2–F1**	2.537(8)
		**Ag2–F2**	2.571(7)
		**Ag2–F1**	2.884(8)
**Bond valence sum**			
**Ag1**	1.94	**Ag2**	1.00

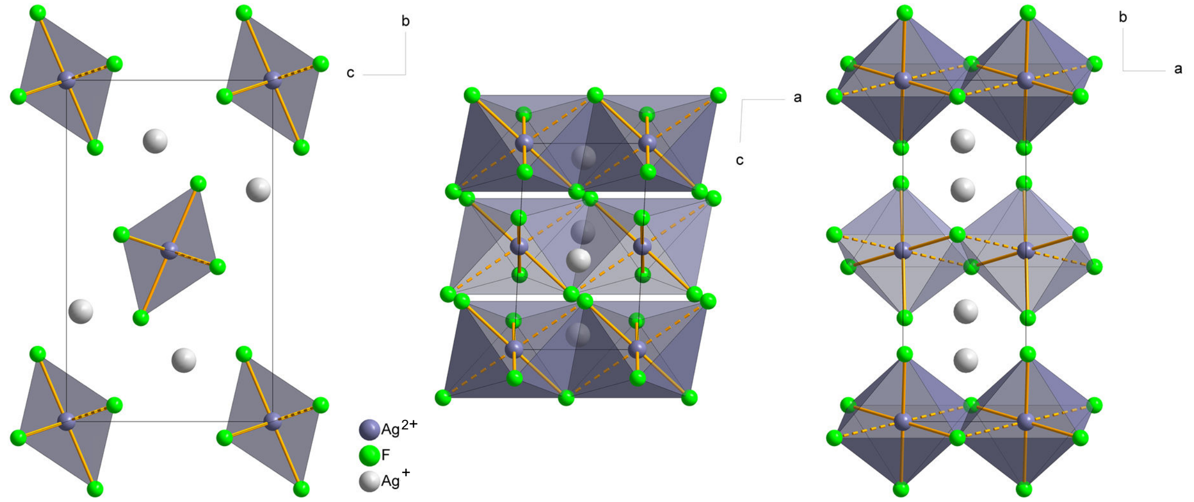

Figure S3. The crystal packing and the unit cell of $Ag^{I}_{2}Ag^{II}F_4$ (Ag_3F_4) crystal structure viewed along the *a*-axis (left), *b*-axis (middle), and *c*-axis (right) of the unit cell.

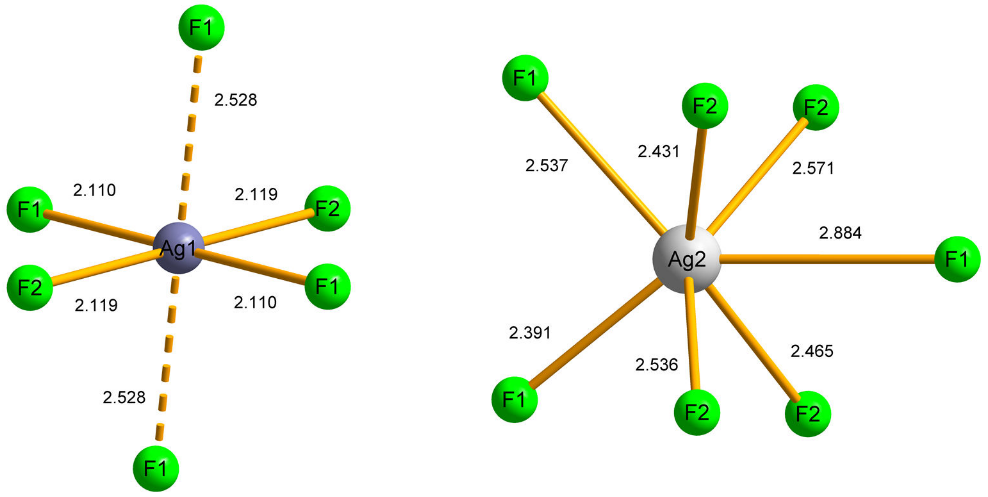

Figure S4. Coordination surroundings with atom distances in Å of silver(II) cation (Ag1) and silver(I) cation (Ag2) in the crystal structure of $Ag^{I}_{2}Ag^{II}F_4$ (Ag_3F_4).

Table S2a. Crystallographic parameters of $Ag^{I}_{2}Ag^{II}F_4$ obtained by the Rietveld refinement of neutron powder diffraction data (λ = 2.41 Å, T = 5 K).

$Ag^{I}_{2}Ag^{II}F_4$	
**Space group**	$P2_1/c$ (no. 14)
**Chemical formula weight** (g/mol)	399.6
Z	2
a*, *b*, *c (Å)	3.5528(4), 9.7931(12), 5.9406(8)
β (°)	92.614(3)
V (Å^3)	206.48(8)
**Ag1** (*x*, *y*, *z*)<br>U_{iso}<br>**Oxidation state**	0, 0, 0<br>0.0025(14)<br>+2
**Ag2** (*x*, *y*, *z*)<br>U_{iso}<br>**Oxidation state**	0.5126(4), 0.1798(2), 0.4299(2)<br>0.0023(12)<br>+1
**F1** (*x*, *y*, *z*)<br>U_{iso}	0.5610(4), 0.4508(3), 0.2665(3)<br>0.0037(14)
**F2** (*x*, *y*, *z*)<br>U_{iso}	0.0072(4), 0.1956(2), 0.1366(2)<br>0.0038(13)
**GOF**	2.084
R_{wp}	0.02352

Table S2b. Interatomic distances and bond valence sum analysis[S7] for silver atoms in $Ag^{I}_{2}Ag^{II}F_4$. The following bond valence parameters were employed: b = 0.37 Å; R_0 = 1.80 Å (Ag^{I}–F) and 1.79 Å (Ag^{II}–F).[S8,S9]

**Interatomic distances** (Å)			
**Ag1–F1** (× 2)	2.0957(15)	**Ag2–F1**	2.373(2)
**Ag1–F2** (× 2)	2.080(2)	**Ag2–F2**	2.4266(19)
**Ag1–F1** (× 2)	2.5259(15)	**Ag2–F2**	2.449(2)
		**Ag2–F2**	2.534(2)
		**Ag2–F1**	2.535(2)
		**Ag2–F2**	2.536(3)
		**Ag2–F1**	2.834(3)
**Bond valence sum**			
**Ag1**	2.06	**Ag2**	1.04

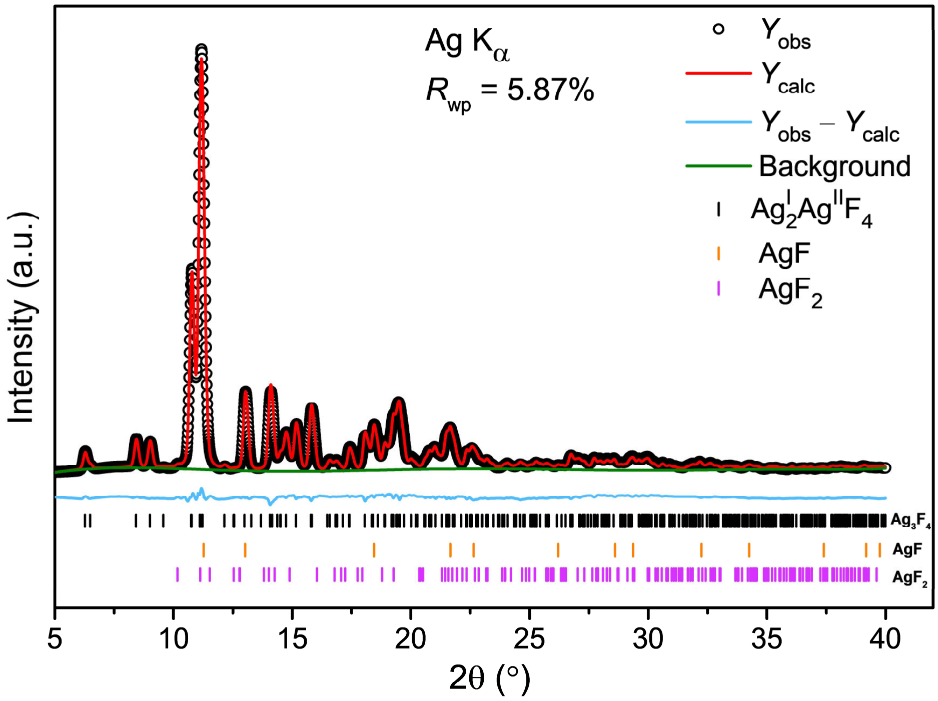

Figure S5. Rietveld refinement profile of the $Ag^I_2Ag^{II}F_4$ sample prepared by solid-state synthesis (laboratory PXRD data, room temperature, Ag Kα radiation, λ = 0.5609 Å). Quantitative analysis: **$Ag^I_2Ag^{II}F_4$: 85.4(2) wt %**; AgF: 14.3(1) wt %; AgF_2: 0.3(4) wt %.

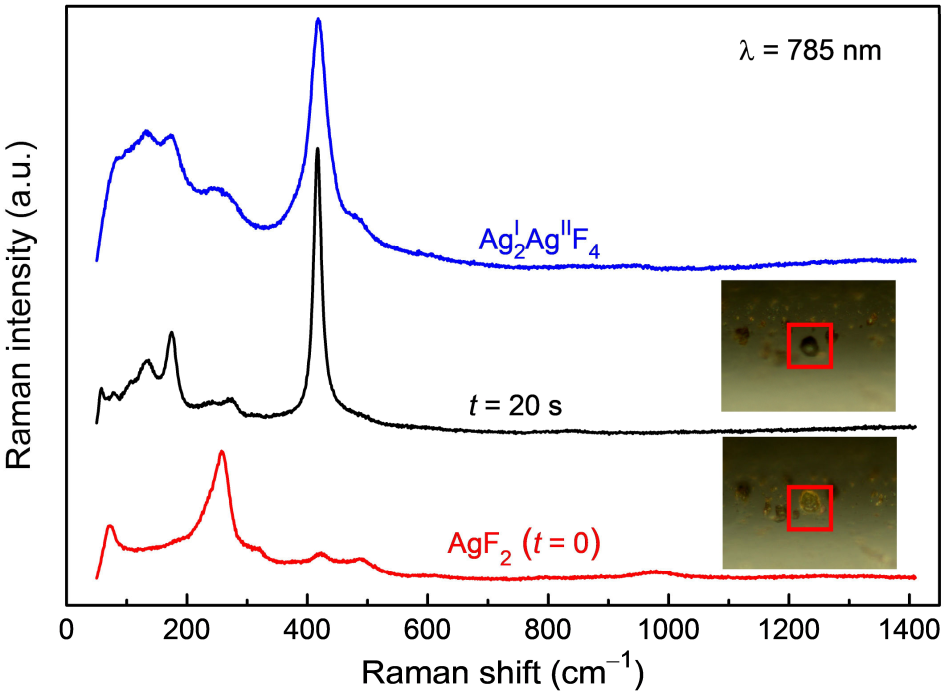

Figure S6. Raman spectra measured using a 785 nm excitation laser with 1 mW power. Bottom Raman spectrum (red) corresponds to AgF_2, the middle spectrum (black) was obtained after AgF_2 was illuminated with green laser (532 nm, 12.5 mW) for 20 seconds, and top spectrum (blue) was obtained on the mechanochemically synthesized $Ag^I_2Ag^{II}F_4$.

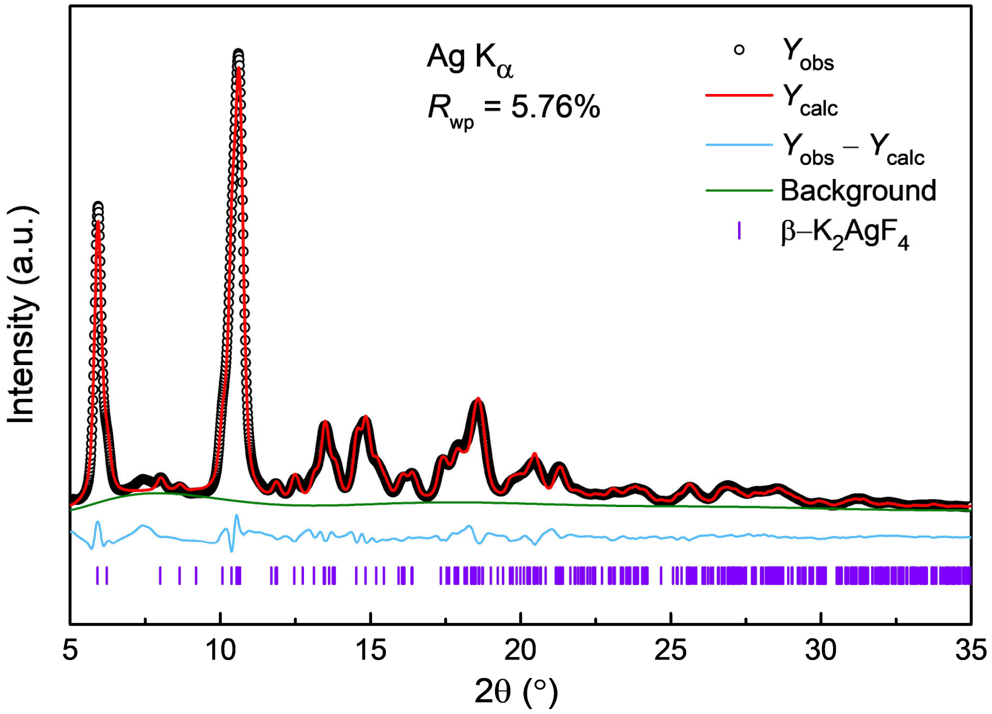

Figure S7. Rietveld refinement profile of the mechanochemically synthesised β-K_2AgF_4 sample (laboratory PXRD data, room temperature, Ag Kα radiation, λ = 0.5609 Å). Note that there is one unidentified peak at around 7.5° 2Θ.

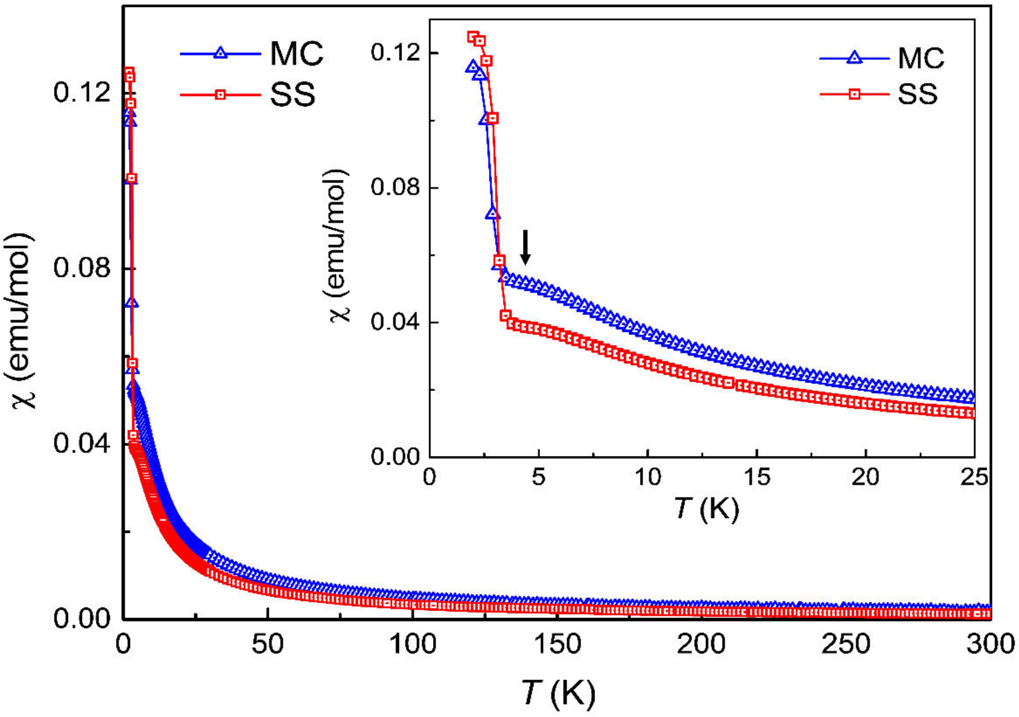

Figure S8. Zero field-cooled curves of temperature-dependent magnetic susceptibility of the $Ag^{I}_2Ag^{II}F_4$ samples prepared by mechanochemistry (MC) and by the solid-state route (SS).

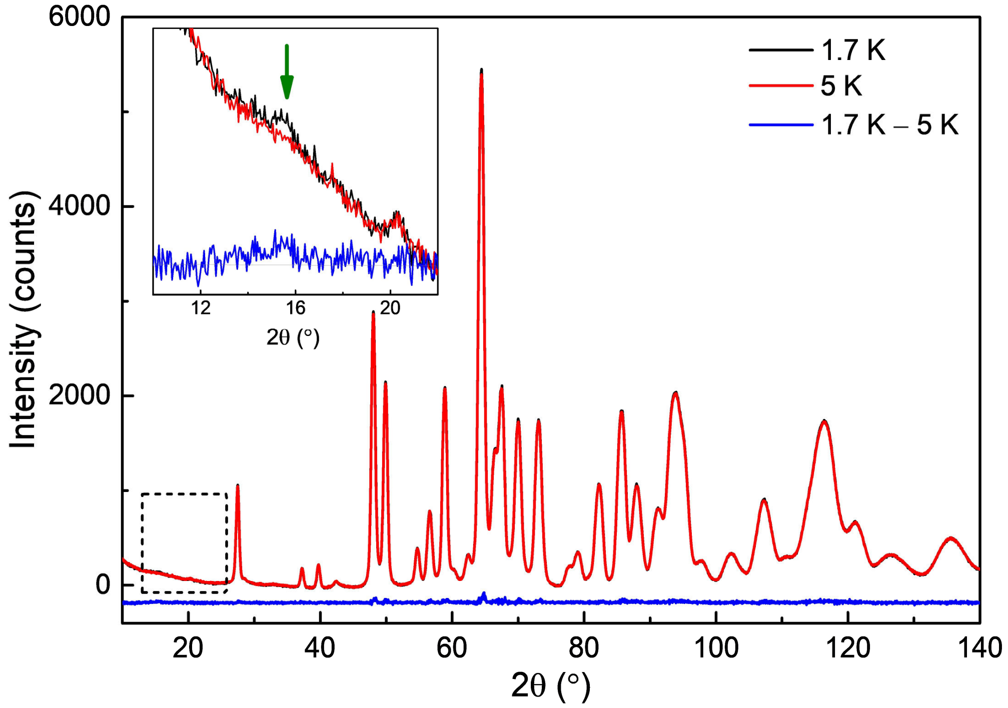

Figure S9. The neutron powder diffraction data of MC $Ag^{I}_2Ag^{II}F_4$ sample measured at 5 and 1.7 K and the difference plot. A magnetic peak at 2θ angle of about 16° can be observed (green arrow in the inset).

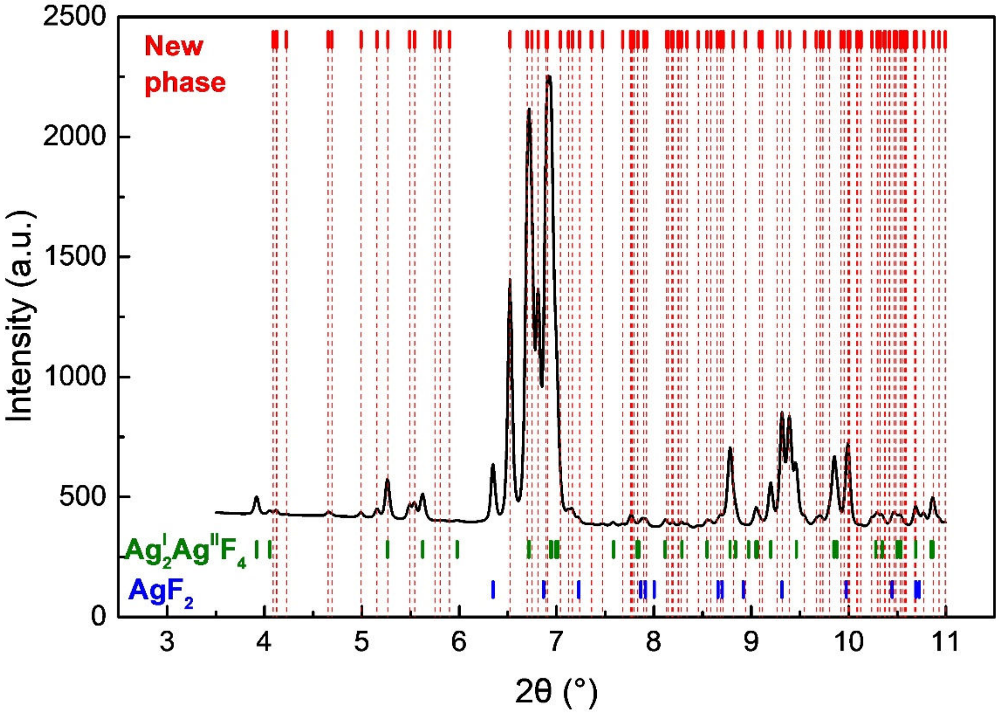

Figure S10. Unit cell indexing (*P*−1 space group) of the synchrotron PXRD data (room-temperature, λ = 0.3497 Å) measured on $Ag^{I}Ag^{II}F_3$ sample prepared by the solid-state synthesis. For clarity, only the range up to 11° 2θ is displayed.

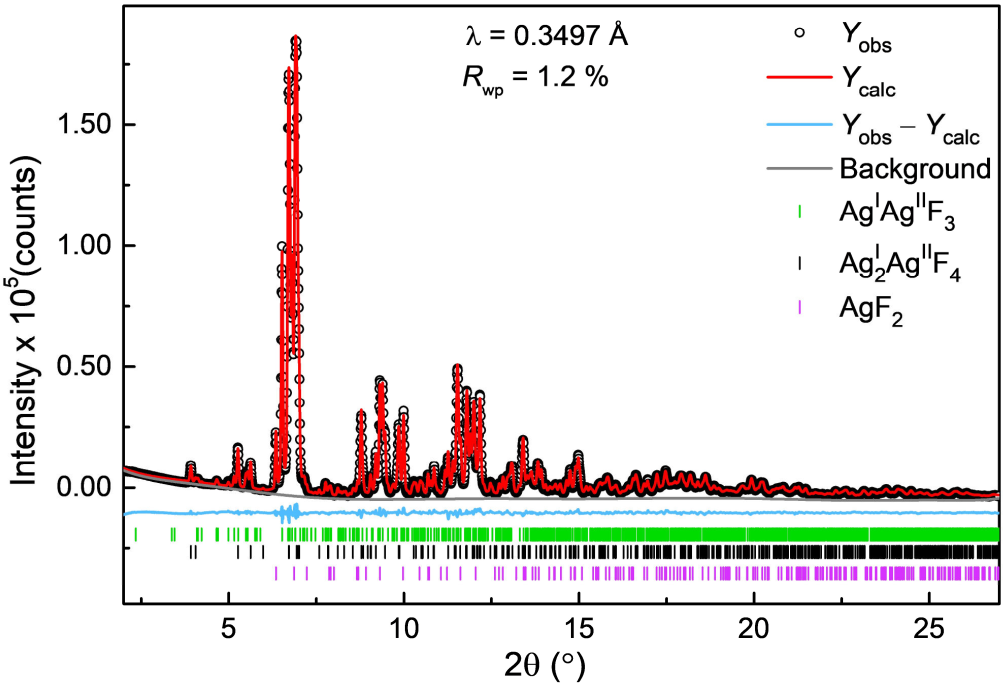

Figure S11. Rietveld refinement profile of the room-temperature synchrotron data for the SS $Ag^{I}Ag^{II}F_3$ sample. Quantitative analysis: $Ag^{I}Ag^{II}F_3$: 57.4(1) wt %; $Ag^{I}_2Ag^{II}F_4$: 39.3(1) wt %; AgF_2: 3.3(1) wt %.

Table S3a. Crystallographic parameters of $Ag^{I}Ag^{II}F_3$ obtained by the Rietveld refinement of room-temperature synchrotron data (λ = 0.3497 Å).

$Ag^{I}Ag^{II}F_3$	
**Space group**	*P*–1 (no. 2)
**Chemical formula weight** (g/mol)	272.73
Z	4
a*, *b*, *c (Å)	5.9577(1), 5.8217(1), 8.5467(2)
**α, β, γ** (°)	91.565(2), 90.644(2), 85.977(1)
V ($Å^3$)	295.582(6)
**Ag1** (*x*, *y*, *z*)<br>U_{iso}<br>**Oxidation state**	0.0515(8), 0.9834(8), 0.2550(5)<br>0.020(2)<br>+1
**Ag2** (*x*, *y*, *z*)<br>U_{iso}<br>**Oxidation state**	0.5501(8), 0.5066(8), 0.2444(5)<br>0.025(2)<br>+1
**Ag3** (*x*, *y*, *z*)<br>U_{iso}<br>**Oxidation state**	0.5, 0, 0<br>0.006(3)<br>+2
**Ag4** (*x*, *y*, *z*)<br>U_{iso}<br>**Oxidation state**	0, 0.5, 0<br>0.005(3)<br>+2
**Ag5** (*x*, *y*, *z*)<br>U_{iso}<br>**Oxidation state**	0.5, 0, 0.5<br>0.009(3)<br>+2
**Ag6** (*x*, *y*, *z*)<br>U_{iso}<br>**Oxidation state**	0, 0.5, 0.5<br>0.009(3)<br>+2
**F7** (*x*, *y*, *z*)<br>U_{iso}	0.064(3), 0.594(3), 0.733(1)<br>0.021(6)
**F8** (*x*, *y*, *z*)<br>U_{iso}	0.212(3), 0.218(3), 0.559(3)<br>0.042(7)
**F9** (*x*, *y*, *z*)<br>U_{iso}	0.204(2), 0.198(2), −0.078(2)<br>0.008(6)
**F10** (*x*, *y*, *z*)<br>U_{iso}	0.310(3), 0.729(3), 0.430(2)<br>0.014(6)
**F11** (*x*, *y*, *z*)<br>U_{iso}	0.291(2), 0.670(3), 0.046(3)<br>0.016(7)
**F12** (*x*, *y*, *z*)<br>U_{iso}	0.562(3), 0.893(3), 0.771(1)<br>0.014(6)
**GOF**	2.159
R_{wp}	0.0120

Table S3b. Interatomic distances and bond valence sum analysis[S7] for silver atoms in $Ag^{I}Ag^{II}F_3$. The following bond valence parameters were employed: b = 0.37 Å; R_0 = 1.80 Å (Ag^{I}–F) and 1.79 Å (Ag^{II}–F).[S8,S9]

**Interatomic distances** (Å)			
**Ag1–F9**	2.409(15)	**Ag3–F12** (× 2)	2.066(11)
**Ag1–F12**	2.473(17)	**Ag3–F9** (× 2)	2.147(13)
**Ag1–F7**	2.508(17)	**Ag3–F11** (× 2)	2.407(16)
**Ag1–F10**	2.560(17)		
**Ag1–F8**	2.607(19)	**Ag4–F11** (× 2)	2.081(15)
**Ag1–F11**	2.843(18)	**Ag4–F9** (× 2)	2.161(13)
**Ag1–F8**	3.072(20)	**Ag4–F7** (× 2)	2.403(11)
**Ag2–F7**	2.341(16)	**Ag5–F10** (× 2)	2.078(16)
**Ag2–F11**	2.430(17)	**Ag5–F8** (× 2)	2.115(16)
**Ag2–F10**	2.451(19)	**Ag5–F12** (× 2)	2.435(11)
**Ag2–F12**	2.464(18)		
**Ag2–F9**	2.745(19)	**Ag6–F7** (× 2)	2.072(16)
**Ag2–F8**	2.772(15)	**Ag6–F8** (× 2)	2.087(11)
**Ag2–F11**	2.806(20)	**Ag6–F10** (× 2)	2.441(16)
**Bond valence sum**			
**Ag1**	0.84	**Ag3**	2.09
**Ag2**	0.97	**Ag4**	2.03
		**Ag5**	2.10
		**Ag6**	2.18

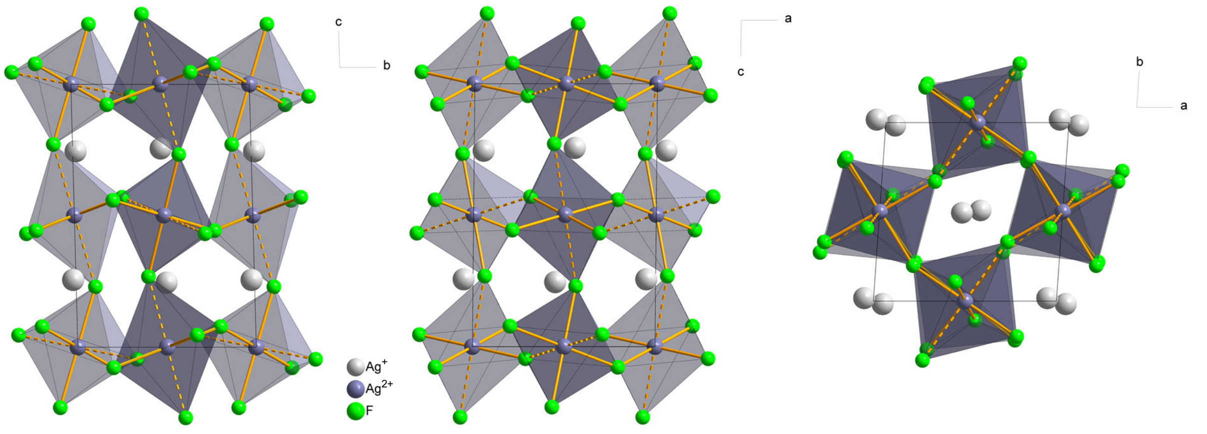

Figure S12. The crystal packing and the unit cell of $Ag^{I}Ag^{II}F_3$ (Ag_2F_3) crystal structure viewed along the *a*-axis (left), *b*-axis (middle), and *c*-axis (right) of the unit cell.

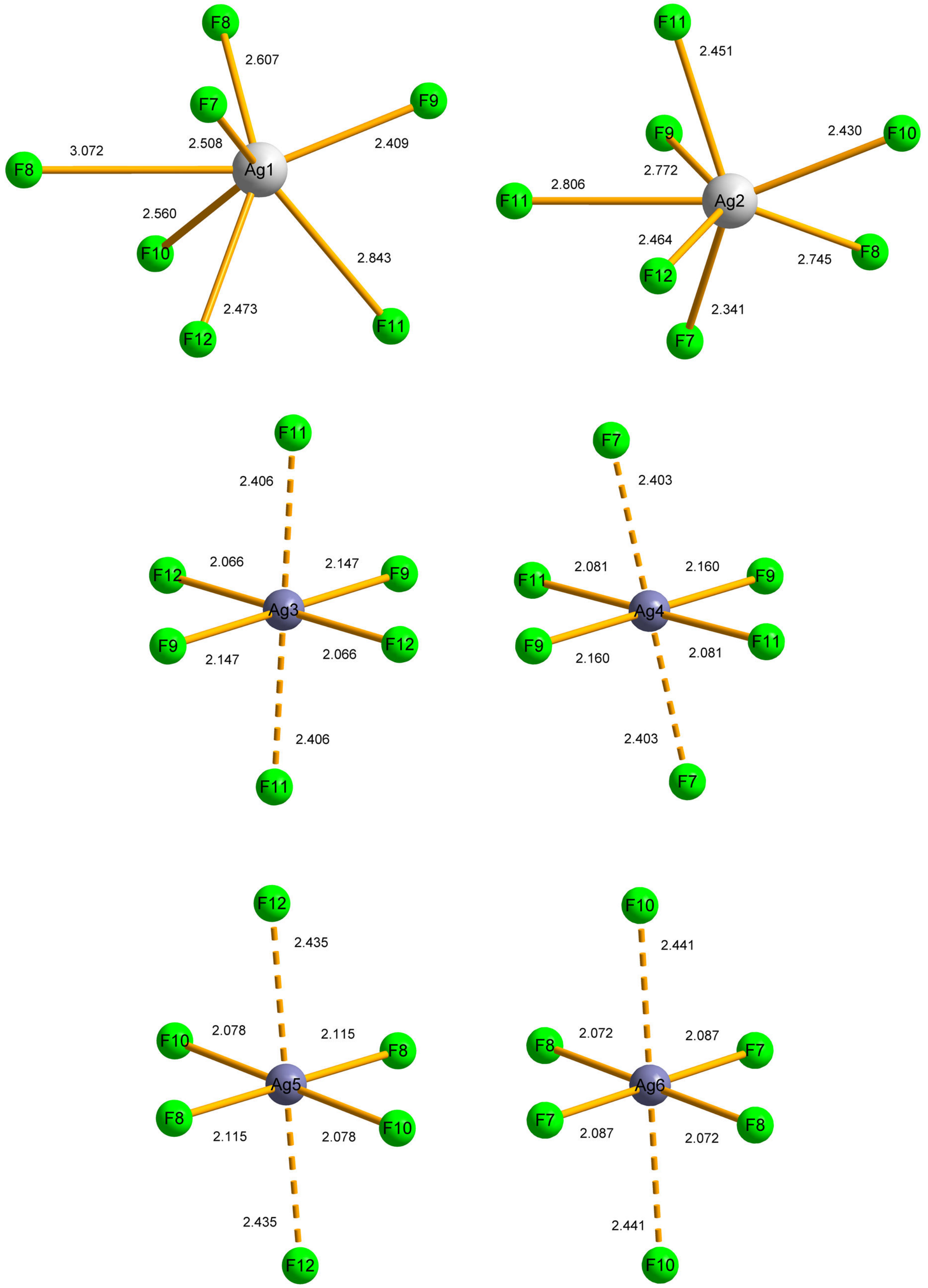

Figure S13. Coordination surroundings with atom distances in Å of silver(I) cations (Ag1, Ag2) and silver(II) cations (Ag3, Ag4, Ag5, Ag6) in the crystal structure of $Ag^{I}Ag^{II}F_3$ (Ag_2F_3).

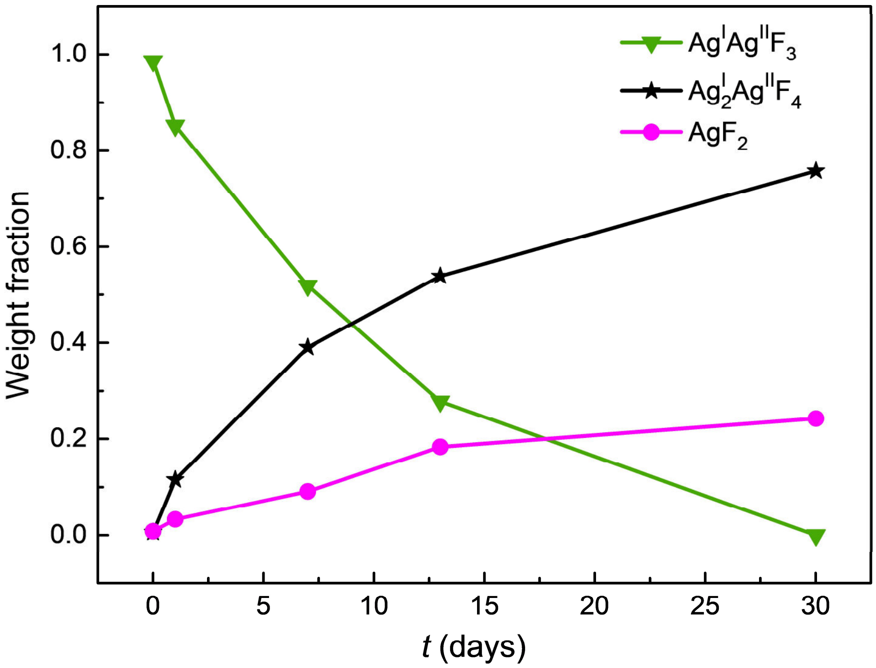

Figure S14. Time-dependent evolution of the phase composition of the mechanochemically (MC) $Ag^{I}Ag^{II}F_3$ sample stored in the glovebox, assessed over a 30-day period. Weight fraction were obtained from Rietveld refinement of the laboratory PXRD data measured at room temperature. The amorphous phase was not accounted in the refinement, which resulted in an overestimation of the crystalline phases. The error bars are smaller than the symbols in the graph.

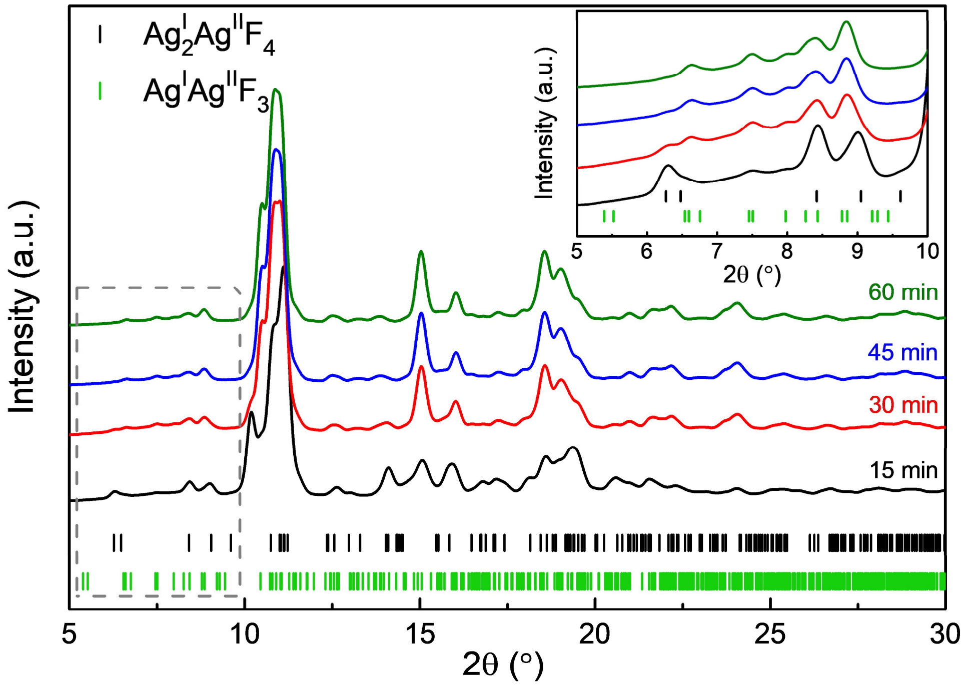

Figure S15. Time-dependent sampling during the mechanochemical synthesis of $Ag^{I}Ag^{II}F_3$. It seems that the $Ag^{I}_2Ag^{II}F_4$ initially forms concomitantly with the $Ag^{I}Ag^{II}F_3$ phase and gets consumed as the reaction proceeds.

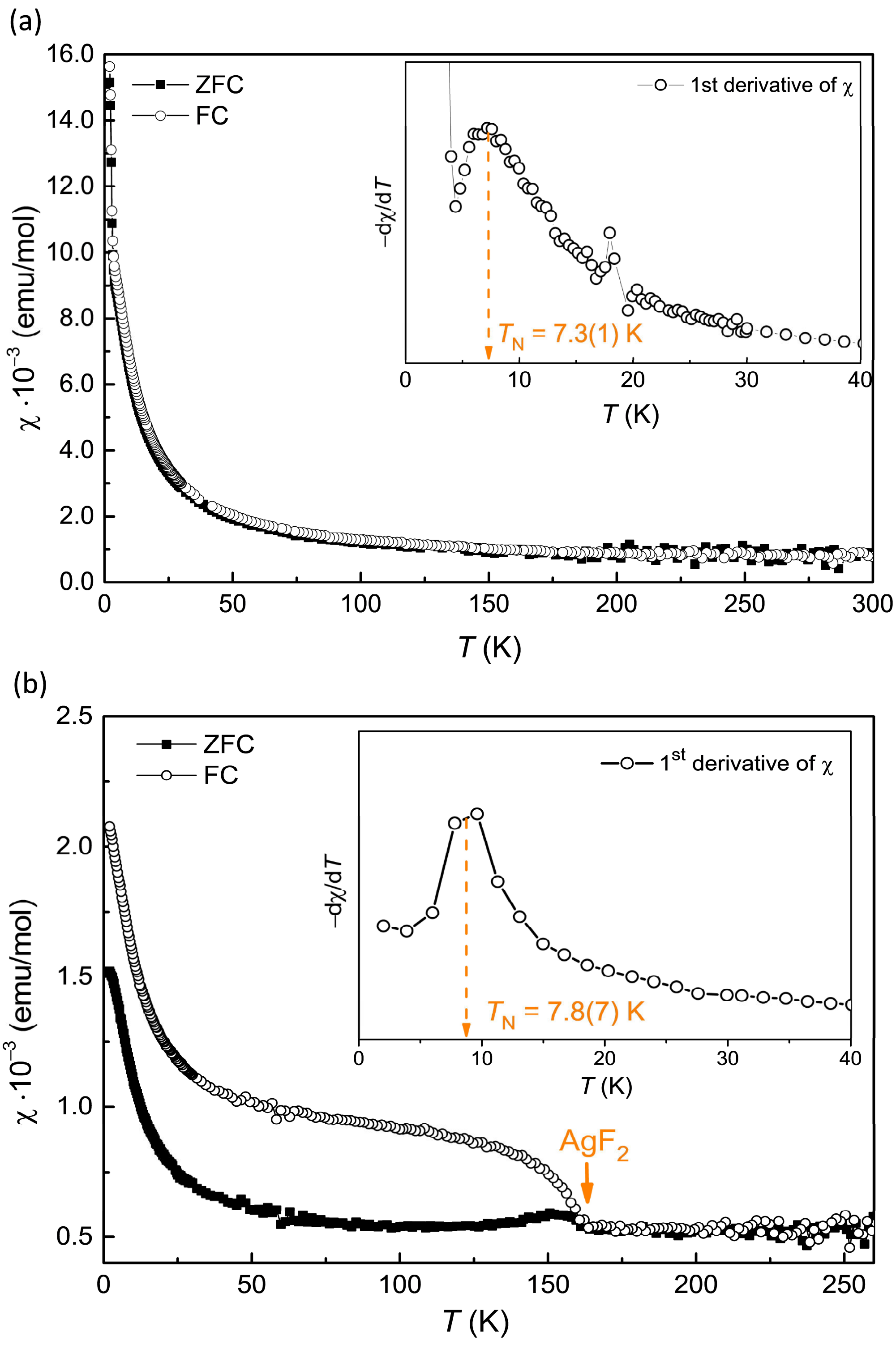

Figure S16. Temperature-dependent magnetic susceptibility, χ, of mechanochemically synthesized $Ag^{I}Ag^{II}F_3$, measured in a magnetic field of 1 kOe. Sample (**a**): $Ag^{I}Ag^{II}F_3$: 78.6(2) wt %; $Ag^{I}_2Ag^{II}F_4$: 21.4(1) wt % (derived from laboratory PXRD). Sample (**b**): $Ag^{I}Ag^{II}F_3$: 96.7 wt %; AgF_2: 3.3 wt % (estimated from χ·*T* data).

SECTION E References

[S1] Wilkinson, J. M.; Blundell, S. J.; Biesenkamp, S.; Braden, M.; Hansen, T. C.; Koteras, K.; Grochala, W.; G.; Barone, P.; Lorenzana, J.; Mazej, Z.; Tavčar, G. Low-temperature magnetism of $KAgF_3$. *Phys. Rev. B* **2023**, *107* (14), 144422. DOI: 10.1103/PhysRevB.107.144422.

[S2] Degen, T.; Sadki, M.; Bron, E.; König, U.; Nénert, G. The HighScore suite. *Powder Diffr.* **2014**, *29* (S2), S13–S18. DOI: 10.1017/S0885715614000840

[S3] Rigaku OD. *CrysAlis^{Pro}*. Rigaku Corporation, Wrocław, Poland, **2023**.

[S4] Toby, B. H.; Von Dreele, R. B. GSAS-II: the genesis of a modern open-source all purpose crystallography software package. *J. Appl. Crystallogr.* **2013**, *46*, 544–549. DOI: 10.1107/S0021889813003531.

[S5] Dragomir, M.; Belak Vivod, M.; Hansen, T. C. Neutron diffraction study of Ag^{2+} based materials. **2023.** Institut Laue-Langevin (ILL). DOI: 10.5291/ILL-DATA.DIR-281

[S6] Kahn, O. *Molecular Magnetism*. VCH Publishers, New York, **1993**.

[S7] Brown, I. D. *The Chemical Bonding in Inorganic Chemistry: The Bond Valence Model*. Second Edition, Oxford University Press, Oxford, United Kingdom, 2016. DOI: 10.1093/acprof:oso/9780198742951.001.0001

[S8] Brese, N. E.; O'Keeffe, M. Bond-valence parameters for solids. *Acta Crystallogr.* **1991**, B47, 192–197. DOI: 10.1107/S0108768190011041

[S9] Brown, I. D. bvparm2020.cif, Accumulated Table of Bond Valence Parameters, (IUCr) Bond valence parameters, http://www.iucr.org/resources/data/datasets/bond-valence-parameters (accessed: August 2024), Brockhouse Institute for Materials Research, McMaster University, Hamilton, ON, Canada.
